# Normalizing and Correcting Variable and Complex LC–MS Metabolomic Data with the R Package pseudoDrift

**DOI:** 10.3390/metabo12050435

**Published:** 2022-05-12

**Authors:** Jonas Rodriguez, Lina Gomez-Cano, Erich Grotewold, Natalia de Leon

**Affiliations:** 1 Department of Agronomy, University of Wisconsin-Madison, Madison, WI 53706, USA; ndeleongatti@wisc.edu; 2 Department of Biochemistry and Molecular Biology, Michigan State University, East Lansing, MI 48824, USA; gomezca5@msu.edu (L.G.-C.); grotewol@msu.edu (E.G.)

**Keywords:** maize, metabolomics, LC–MS, signal drift, data normalization

## Abstract

In biological research domains, liquid chromatography–mass spectroscopy (LC-MS) has prevailed as the preferred technique for generating high quality metabolomic data. However, even with advanced instrumentation and established data acquisition protocols, technical errors are still routinely encountered and can pose a significant challenge to unveiling biologically relevant information. In large-scale studies, signal drift and batch effects are how technical errors are most commonly manifested. We developed pseudoDrift, an R package with capabilities for data simulation and outlier detection, and a new training and testing approach that is implemented to capture and to optionally correct for technical errors in LC–MS metabolomic data. Using data simulation, we demonstrate here that our approach performs equally as well as existing methods and offers increased flexibility to the researcher. As part of our study, we generated a targeted LC–MS dataset that profiled 33 phenolic compounds from seedling stem tissue in 602 genetically diverse non-transgenic maize inbred lines. This dataset provides a unique opportunity to investigate the dynamics of specialized metabolism in plants.

## 1. Introduction

Metabolomics concerns the study of small molecular compounds or metabolites (<1500 Da) and is essential for advancements in metabolism research in biological systems [1]. The metabolome is influenced by complex interactions between the genome, proteome, and transcriptome with the environment [2], and evoking changes to the metabolome and the associated effects on metabolic pathways has direct applications to pharmacology, drug development, and improvements to plant productivity, composition, and resilience [3,4]. Maize is among the most productive and economically important crops, with an annual production of over 700 million metric tons globally [5]. Many tools and technologies have facilitated previous maize improvement efforts, and future improvements will benefit from metabolomics-based research [6]. In particular, valuable insights are likely to come from larger scale metabolomic experiments and evaluations.

A metabolomic analysis involves identifying and/or quantifying many metabolites simultaneously either by targeted (metabolite identity known) or untargeted (metabolite identity unknown) methods [1]. Liquid chromatography coupled with tandem mass spectrometry (LC–MS) is currently the dominant technique used in biological research due to its high sensitivity and selectivity [7,8]. However, metabolic profiles from extracts obtained from genetically identical individuals, or even from the same biological sample can have significant variability, the sources of which can be traced to extraction efficiency, changes in the injection volume, inlet interference contamination, column contamination (due to the complex matrices), or drift in ionization efficiency [8]. Despite taking appropriate actions during the data acquisition phase to minimize unwanted sources of variation, obtaining repeatable measurements across different instruments, and even on the same instrument, remains a challenging task. Furthermore, these challenges can be compounded in large-scale studies where LC–MS runs are often split into several analytical batches, each potentially containing a unique combination of technical variability [9].

Combining data across batches in large-scale studies is imperative for increasing the statistical power of downstream analyses and interpretation. Therefore, several strategies have emerged to capture and correct for systematic errors in metabolomics data [10]. The most robust methods rely on regularly interspersed quality control (QC) samples included within and between batches [11], which are meant to capture temporal signal drift trends and detect any additional technical errors. QC data points can then be used to apply a computationally efficient correction to the entire dataset, for instance, with a QC-Robust Spline Correction (QC-RSC) [12]. However, there is no universally applicable approach to predetermine QC sample composition or the frequency at which QC samples should be included along the LC–MS run sequence; these are typically influenced by practical and experiment-specific considerations. While a high frequency of QC sample inclusion may be useful for thoroughly capturing systematic errors, these samples occupy LC–MS run slots that might otherwise be allocated to experimental samples, increasing the size of the experiment and subsequently increasing the opportunity for error. To this end, non-QC-based correction approaches have been attempted [8,13,14]. However, the benefits from increased experimental throughput come at the expense of a potentially reduced ability to detect and correct for systematic errors.

Here, we present pseudoDrift, a new R package that combines the beneficial aspects of QC- and non-QC-based technical error correction methods. pseudoDrift relies on a training and testing procedure to estimate non-observed QC data points (pseudoQC) from metabolomics data with partial QC (trueQC) representation. pseudoDrift is not a correction method per se, but rather offers a strategy for estimating pseudoQC reference points that can be used to make data corrections using other already existing methods. By default, pseudoDrift uses the QC-RSC correction method [12]. However, the non-adjusted data containing pseudoQC estimations are also returned and can be exported if alternative correction methods are preferred by the user. Additional functionality of pseudoDrift comes in the form of simulation and outlier detection capabilities. Compared to currently available LC–MS simulation tools [15,16,17,18], which aim to simulate raw data that need to subsequently be processed before generating manipulatable peak matrices, pseudoDrift allows for direct simulation of peak matrices based on metadata gathered from online databases such as the MassBank of North America [19]. The outlier detection method implemented in pseudoDrift relies on absolute differences between sample replicates to generate an expected tailed distribution, which is used to identify potential outliers. The pseudoDrift package is publicly available at https://github.com/jrod55/pseudoDrift (accessed on 11 April 2022). In this study, the main objectives were to demonstrate the utility of pseudoDrift using simulated data and to apply the workflow to a newly generated large-scale maize seedling LC–MS phenolic profiling dataset. Although the metabolites extracted and analyzed were from maize, the development and assessment of this tool are applicable to any specific organism. We selected maize because of its importance as a crop and the availability of a well-studied diversity panel [20,21].

## 2. Results and Discussion

### 2.1. Simulating Data with pseudoDrift

**Figure 1:**
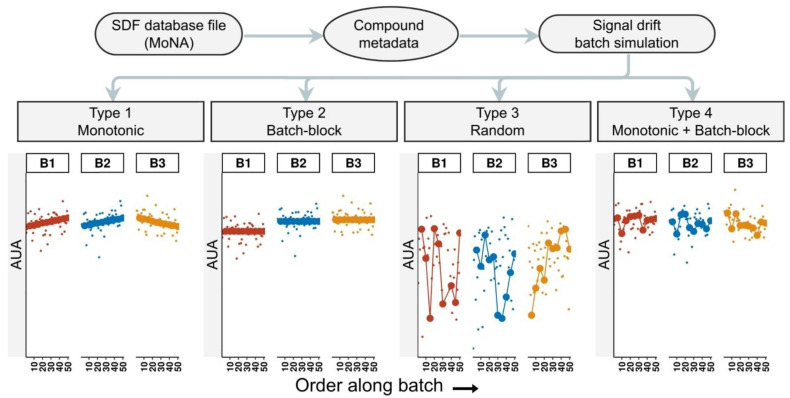
Simulated data produced by the ‘simulate_data()’ function. For each compound queried in the user provided structure-data file (SDF), a simulated peak area matrix with arbitrary units of area (AUA) is returned, along with four additional matrices, each with a different signal drift effect type applied. SDF files are available through the MassBank of North America (MoNA). The larger points in each plot represent the simulated QC samples, and smaller points represent non-QC simulated data points.

To compare each of the analysis workflow functions from pseudoDrift with existing approaches, we used data produced with the ‘simulate_data()’ function, assuming three batches with sample sizes of 100, 200, and 300, respectively (Figure S1). We set the QC frequency to have each 25th sample represent a trueQC, and used the default effect severity settings (slope and batch magnitude set to 1.25). The simulated data corresponded to the phenolic compound tricin, which we queried using the MoNA [19] accession ID FIO00738. From the simulated data output, we retained the type 4 signal drift effect data for analysis, and as a control, the data without any signal drift effects (see Materials and Methods). As a reference, and to depict the inputs and outputs of ‘simulate_data()’, we included a visual representation of a simple example of three batches that we did not use in our analysis (Figure 1).

### 2.2. Performance Evaluation of the pseudoDrift Analysis Workflow

**Figure 2:**
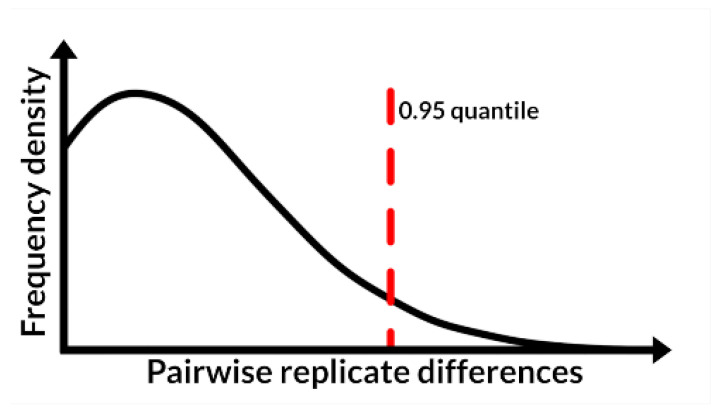
Visual representation of the outlier detection approach implemented in the ‘pw_outlier()’ function. Given a peak area matrix for a particular compound or feature, all pairwise differences between sample replicates are computed. The distribution of these differences is expected to be positively skewed, with values surpassing a given quantile threshold (0.95% shown) marked as potential outliers.

For outlier detection, we compared ‘pw_outlier()’ to the commonly used 1.5*Interquartile Range (IQR) method, and a formal statistical testing approach with an iterative evaluation of the Grubbs test statistic [22] with a 0.05 type 1 error rate. With ‘pw_outlier‘, we used the default 0.95 quantile threshold (Figure 2), and we observed the expected tailed distribution in each batch of the simulated data (Figure S2).

Between outlier detection methods tested, ‘pw_outlier()’ identified a total of 24 potential outliers, IQR detected 7, and the iterative Grubbs test identified a single point (Figure S3). Although ‘pw_outlier()’ detected the largest number of possible outliers, they were not all necessarily at the extremes of the peak area distribution. The distinguishing feature of ‘pw_outlier()’, compared to the two conventional outlier detection methods tested, was that it accounted for the experimental unit (set of three observations) as opposed to treating the distribution of all observations as a whole. This was particularly relevant given that extreme observations, when consistent between replicate measures, could represent biological anomalies that may warrant further investigation. Treating all samples as a whole and basing outlier detection on their distribution would not necessarily have captured the within experimental unit variability as ‘pw_outlier()’ did. A noteworthy characteristic of ‘pw_outlier()’ is that the number of potential outliers returned will always be a function of the quantile threshold set. In our simulated data, the 24 potential outliers represented 5% (1-quantile threshold) of non-QC observations from each batch. Therefore, the user should take this into consideration when applying ‘pw_outlier()’ and interpreting the results.

**Figure 3:**
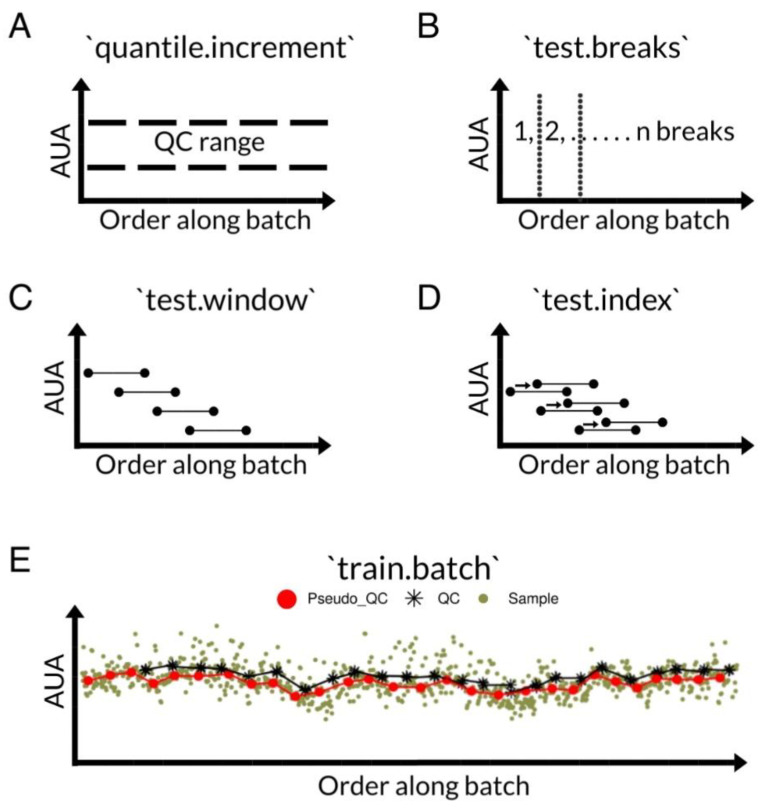
The parameters (**A**–**D**) optimized for ‘pseudo_sdc()’ that minimize the criteria set by the user. The parameter name is given in the title of each plot, and all computations are made using arbitrary units of area (AUA). By default, the mean squared error is minimized between estimated pseudoQC and true QC samples in the training batch (**E**).

We assessed the performance of ‘pseudo_sdc()’ by using the simulated data batch with the largest sample size as the training batch and then comparing the coefficient of determination (R^2^) and root mean squared error (RMSE) from 10-fold cross-validation between signal drift corrected data and the control. Here, the comparison we made was between using either pseudoQC or trueQC samples to correct for signal drift with the QC-RSC [12] method. As a reference, we included a visual representation of parameters optimized by ‘pseudo_sdc()’ and an example of how the process was carried out (Figure 3).

Gauging the performance of ‘pseudo_sdc()’ solely on its ability to reduce the variability among the simulated trueQC samples would have underestimated the ability of pseudoQC samples to capture and correct for signal drift (Figure S4). However, the benefit of using simulated data was that each observation effectively served as a QC sample since we knew the originally simulated value. Regressing the signal drift corrected data on the originally simulated values, we found that using pseudoQCs to correct the data resulted in a correction (R^2^ = 0.7404; RMSE = 3810) which was on par with the performance of a trueQC-based correction (R^2^ = 0.7499; RMSE = 3744) (Figure S5). Our data-driven approach, which took advantage of the data variability while training a model on trueQCs as anchors in the training batch, also reduced systematic bias in how the signal drift correction was applied. The trueQC correction more effectively corrected a portion of batch 3 in the simulated data compared to other batches (Figure S5), whereas the pseudoQC correction appeared to have an equal performance across batches (Figure S5). The bias reduction with the pseudoQC correction was likely due to the differences between how trueQC and pseudoQC samples captured the signal drift trend. The trueQC samples were independent of the data as a whole and were able to capture sharp changes in signal drift, while pseudoQC provided a more general representation of the trend and captured more subtle signal drift patterns with information from the data variability.

### 2.3. Maize LC–MS Phenolic Data Analysis with pseudoDrift

Prior to applying the pseudoDrift analysis workflow to the normalized maize LC–MS dataset consisting of peak areas for 33 phenolic compounds (Table S1), we identified five compounds (apigenidin, dihydrokaempferol, luteolin 7-*O*-glucoside, syringic acid, and syringol) with limits of detection (LOD) threshold values greater than 25% of all experimental samples across sub-batches. The LOD for each compound varied by sub-batch (Table S1), and, therefore, so did the proportion of experimental samples above the LOD. To avoid a large proportion of missing data for apigenidin, dihydrokaempferol, luteolin 7-*O*-glucoside, syringic acid, and syringol, they were completely excluded from downstream analyses. The remaining compounds were independently analyzed with the ‘pw_outlier()’ function with default arguments. This identified the top 5% of observations per batch as possible outliers. Importantly, with the conservative action of omitting all observations identified with ‘pw_outlier()’, no single inbred line was removed completely from the data. The ‘pw_outlier()’ cleaned data were subsequently processed with ‘pseudo_sdc()’ with batch 4 used as the training batch. The optimal parameters to estimate pseudoQC samples were determined for each compound (Table S2) and used to apply the signal drift correction across batches (File S2). Each compound had distinct signal drift patterns, although the batch-to-batch effect was substantially more pronounced for some compounds. For example, caffeic acid and 4-chlorogenic acid in batch 2 were considerably lower than in other batches (File S2). However, since ‘pseudo_sdc()’ calculated pseudoQC samples based on quantiles determined in the training batch, we were able to capture this batch-to-batch effect along with the signal drift trends.

**Figure 4:**
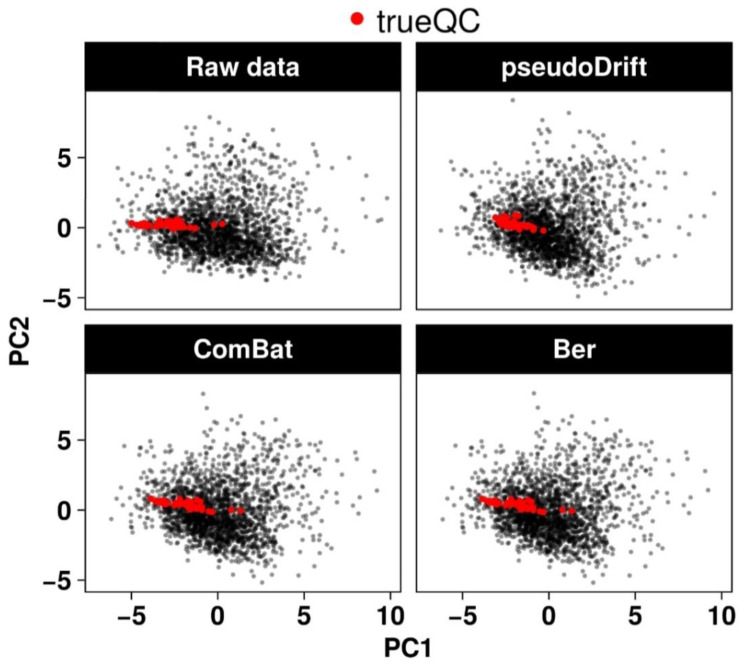
Comparison of different non-QC-based corrections applied to the maize LC–MS peak area matrix. The correction method and resultant peak area matrix used to calculate the first two PCs is labeled on each respective bi-plot. The maximum distance (maxDist) represents the largest distance between any two trueQC points in the bi-plot.

With existing QC-based signal drift and batch correction methods such as QC-RSC [12], we would have been restricted to analyzing the data from batch 4 alone since it was the sole batch with trueQC samples represented (Table 1). Compared to the two non-QC-based methods tested, combatting batch effects (ComBat) [23,24] and batch effect removal (ber) [25], pseudoDrift substantially reduced the maximum distance between any two trueQC points when plotted along the first and second principal components (PCs) (Figure 4). Thus, suggesting an overall improved correction across compounds.

**Figure 5:**
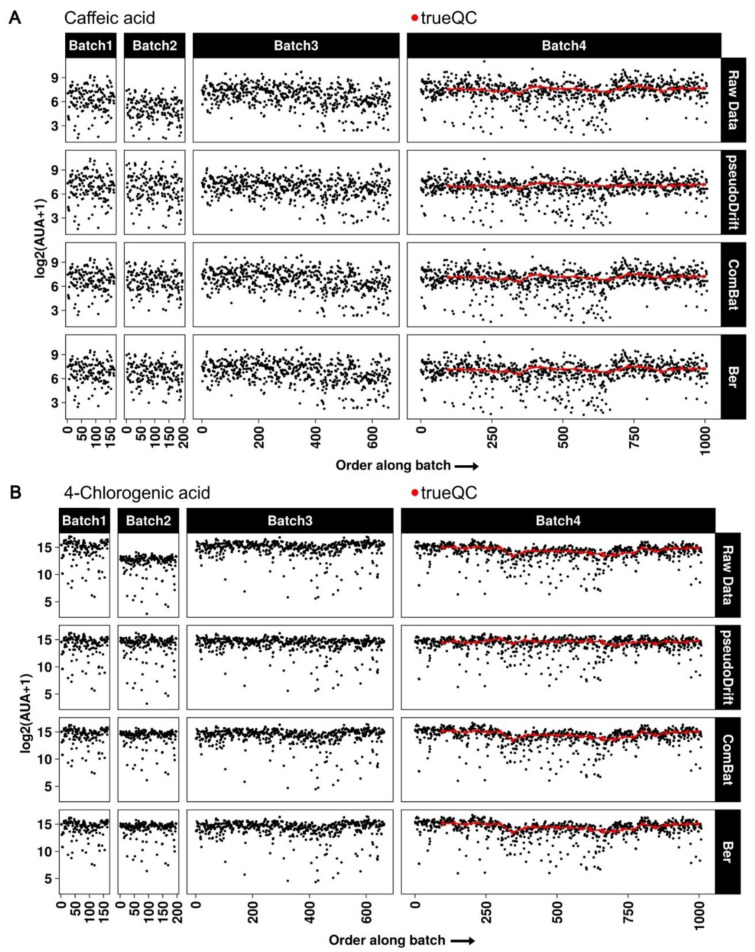
Batch-to-batch and intra-batch signal drift effects before and after correction with different non-QC-based corrections. Plotted are the log2 transformed arbitrary units of area (AUA) for caffeic acid (**A**) and 4-chlorogenic acid (**B**) before correction (raw data) and after correction with each labeled approach.

Independently, even for compounds with severe batch-to-batch effects, such as caffeic acid and 4-chlorogenic acid (File S2), there were no major differences in the inter-batch corrections between pseudoDrift and the ComBat and ber methods (Figure 5). However, as demonstrated by a flatter line among trueQC points in batch 4, pseudoDrift performed best at simultaneously correcting for intra-batch signal drift effects as well. Together, these results highlight the improvements to signal drift and batch corrections, which pseudoDrift achieved by coalescing QC and non-QC approaches into a new correction method.

## 3. Materials and Methods

### 3.1. Plant Material and Experimental Design

We grew a set of 602 genetically diverse maize inbred lines in a controlled environment room under high-intensity light emitting diode (LED) lights at the Wisconsin Crop Innovation Center. Our experiment consisted of a completely randomized design (CRD), with three replications per inbred line, with independent randomization applied per set of 602 lines. We recorded instances where seeds failed to germinate in any of the first three replicates and re-planted these in a fourth replication. In all replications, we sowed seeds into 32-cell flats and hand watered them every other day for the first 7 days. On the 8th day, we transitioned to a daily watering with an automated flood fertigation watering system, which was programmed to submerge the 32-cell flats for 5 min per day. A total of 21 days passed from planting to harvest. During the growing period, we maintained the temperature at 28 °C and artificially controlled the photoperiod by supplying 16 h of light followed by 8 h of darkness. At harvest, we used 15 mL conical Falcon tubes to collect the basal 6 cm of seedling stem tissue and immediately placed samples into liquid nitrogen prior to lyophilizing.

### 3.2. Reagents for Stock and Working Solutions

We procured the reagents used in this study from Sigma-Aldrich (Burlington, MA, USA) Cayman Chemical (Ann Arbor, MI, USA), Indofine chemical (Hillsborough, NJ, USA), and ChromaDex (Irvine, CA, USA) except apimaysin, maysin, and rhamnosylisoorientin, which were provided by Michael McMullen (USDA-ARS) and Maurice Snook (Iowa State University), and when performing dilutions, we used ultrapure (>18 Ω) water generated through a Milli-Q system. The metabolites we profiled included 33 phenolic compounds with available chemical standards (Table S1). For each compound, we prepared 1 mM stock solutions by reconstituting them in 80% (*v*/*v*) HPLC grade methanol or 100% dimethyl sulfoxide (DMSO). We prepared a pooled mixture containing all 33 standards, each at 1 μM, and through serial dilution produced samples with concentrations between 1000 nM and 1.7 nM that we used as external standards. Following the same procedure, but with a final concentration of 50 nM, we prepared an internal standard (8-prenylnaringenin). For sample preparation, we used an extraction solvent consisting of 80% (*v*/*v*) HPLC grade methanol and 0.1% (*v*/*v*) formic acid.

### 3.3. Preparation of Stem Tissue Extracts and QC Samples

The sample preparation occurred in four separate batches, each including a different number of experimental samples (Table 1). Batch 1 was the smallest with 165 samples, followed by batch 2 with 198 samples, batch 3 with 663 samples, and batch 4 with 1008 samples. We homogenized the maize seedling stems using liquid nitrogen and PVC tubes containing a metal bead which a paint shaker (5G-HD Harbil 5-Gallon Shaker model 37600) agitated for 2 min at 60 Hz. To avoid cross-contamination, we washed the PVC tubes and metal beads with distilled water and soap between samples. We then transferred a ~50 mg subset of the homogenized plant material to a 2 mL Eppendorf tube and combined it with the extraction solvent. Batch 4 included the 8-prenylnaringenin internal standard, which was added at the same time that we combined the extraction solvent and the plant material. Our extraction protocol consisted of a 12 h incubation at 4 °C, followed by reconstitution by vortexing for 20 s, centrifugation for 5 min at 15,000× *g* at room temperature, and recovery of the supernatant for analysis by LC–MS. We prepared a QC sample from 100 randomly selected samples and included it in the analysis for batch 4 alone.

### 3.4. LC–MS Data Acquisition

**Figure 6:**
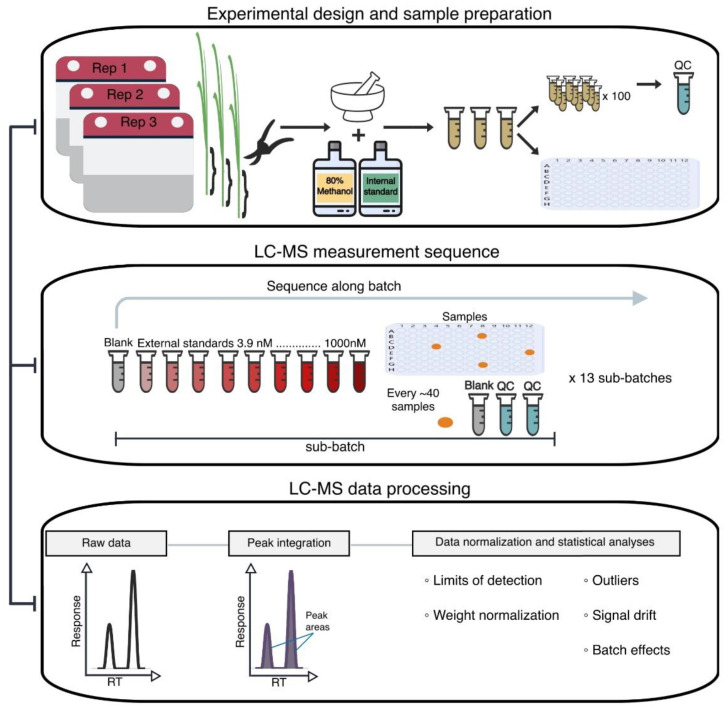
Experimental design, data acquisition, and data processing steps. Samples labeled as QC represent a pooled mixture from 100 randomly selected experimental samples. A representation of the LC–MS measurement sequence for each batch and sub-batch during the data acquisition phase is shown. During data processing, the depiction represents the instrument response as a function of the retention time (RT).

The instrument used for data acquisition was a Waters ACQUITY TQD Tandem Quadrupole UPLC/MS/MS (Waters Corporation, Milford, MA, USA). We created a 10 min targeted multiple reaction monitoring (MRM) method for detecting 33 phenolic compounds (Table S1) and ran samples in accordance with their corresponding preparation batch. The method was based on a modification of the MRM method previously described [26] Within batches, we designated samples to sub-batches based on the preceding external standards set (Figure 6). Across the four batches, we ran a total of 13 external standards sets. For each inbred maize line, we ran the three biological replicates consecutively of one another (e.g., Line 1 rep1, then Line 1 rep2, Line 1 rep 3, Line 2 rep1, etc.). While in the queue, samples and external standards remained at −10 °C in an autosampler. The instrument used a 10 μL injection volume, and the liquid chromatographic separation occurred at 30 °C using a reverse phase Waters Symmetry C18 column (4.6 × 75 mm; 3 μm) with a Symmetry C18 prep-column (3.9 × 20 mm; 5 μm) (Waters Corporation, Milford, MA, USA). We integrated peak areas for all compounds using MassLynx (v 4.2) with vendor-specific data files (.raw) to produce the raw peak area matrix.

### 3.5. PseudoDrift Workflow

The pseudoDrift R package consisted of three main functions, including ‘simulate_data()’, ‘pw_outlier()’, and ‘pseudo_sdc()’. We wrote these functions to run independently of one another, although here we defined the analysis workflow as applying the ‘pw_outlier()’, and ‘pseudo_sdc()’ functions in sequential order.

We wrote the ‘simulate_data()’ function to accept a structure-data file (SDF) as input, such as those obtained from the MassBank of North America (MoNA) [19], and to return the queried compound metadata as output, along with a simulated peak area matrix, and four distinct signal drift and batch effect types applied to the simulated data. The simulated peak area patterns among QC samples were used to define the four effect types. A monotonic increase or decrease was characteristic of a type 1 effect, a type 2 effect described changes in magnitude occurring between batches, a type 3 effect was random, and a type 4 effect consisted of a combination of type 1 and type 2 effects and represented what is most commonly encountered in metabolomics datasets. To provide user flexibility and ensure reproducible simulation results, we included arguments for seed setting, QC frequency, batch size, and effect type severity.

The first analysis function we developed was ‘pw_outlier()’, which served as an outlier detection method to accommodate common features of metabolomic data, including skewed distributions and limited biological or technical replication. Our approach relied on assessing pairwise absolute differences within and between replicate measures of samples. To illustrate, we considered a hypothetical metabolite or feature and a single sample with three biological replicates. The ‘pw_outlier()’ function computed the pairwise differences between replicates as |rep1-rep2|, |rep1-rep3|, and |rep2-rep3| and then extended this computation to each sample within a given batch. This generated a distribution of pairwise differences, which we assumed to be positively skewed with observations at the upper tail representing potential outliers. The default threshold, which we set for ‘pw_outlier‘ to use, was the 0.95 quantile of all sample pairwise differences in a batch. We included arguments allowing the user to have flexibility over the quantile threshold and grouping factor used for calculations.

We developed the second analysis function, ‘pseudo_sdc()’, to work with a variable representation of trueQC samples across batches. Using data corresponding to the batch where trueQC samples were most represented, or the batch designated as the training batch, ‘pseudo_sdc()’ optimized four parameters that were used to calculate pseudoQC points that effectively captured the signal drift pattern in the training batch. To effectively capture signal drift in this context meant to minimize the criterion set by the user, which, by default, we set to use the mean squared error (MSE) between the trueQC samples and the estimated pseudoQC points in the training batch. The four parameters, which ‘pseudo_sdc()’ optimized, concerned the range of values used in the calculation (quantile.increment), the number of equally sized breaks to divide the batch into (test.breaks), the window size to calculate a rolling median over (test.window), and the positional offset (test.index) of pseudoQCs, relative to test.window. With the optimized parameters from the training batch, ‘pseudo_sdc()’ applied the same parameters to the remaining batches to estimate the pseudoQC points from the data variability. We integrated the QC-RSC [12] method into ‘pseudo_sdc()’ with an auxiliary function from the pmp R package [27] to perform the data correction with pseudoQC samples in lieu of the trueQC samples.

To illustrate the functionality of pseudoDrift, we included a tutorial (File S1), where we walked through each of the analysis functions of pseudoDrift. In the tutorial, we used several additional R packages, including ChemmineR [28] for SDF file indexing, ggpubr [29] and cowplot [30] for plotting, data.table [31] and tidyverse [32] for data manipulation, and caret [33] for regression modeling.

### 3.6. LC–MS Data Normalization and Processing with pseudoDrift

We set the limits of detection (LOD) for each phenolic compound as three times the peak area of the blank (extraction solvent alone) and established the thresholds on a per compound and sub-batch basis, with the blank reference value for each compound calculated as the mean peak area across blank samples within the corresponding sub-batch. If cumulatively across sub-batches more than 25% of samples were below the respective compound LOD thresholds, we removed the compounds from the data matrix and completely excluded them from the downstream analyses. Rather than using absolute values, we opted for relative peak area values to ensure compounds were uniformly analyzed, including those that accumulated at high levels in maize stem tissues (outside the upper range of external standards). We normalized the data by the weight of each sample to provide arbitrary units of area (AUA) and removed blanks and external standards from the data matrix prior to analyzing with the pseudoDrift workflow. When applying the analysis workflow, we processed one compound at a time, first with ‘pw_outlier()’, then with ‘pseudo_sdc()’ using batch 4 as the training batch to estimate pseudoQC samples across all batches, and to correct for signal drift and batch effects in the data. We tested two additional non-QC correction methods, specifically the ComBat [23,24] and ber [25] methods implemented in the dbnorm R package [14], and compared the data corrections based on the maximum distance of trueQC points (maxDist) along the first two PCs calculated from the full peak area matrix. A smaller maxDist indicated trueQC samples had less variability, and thus, a better correction of technical errors.

## 4. Conclusions

The number and impact of MS-based metabolomics studies in the biological sciences are likely to rise as methods improve and accessibility to instrumentation by researchers increases. This is particularly true for systems biology, which now has a plethora of complementary omics tools available to investigate previously unexplored areas of research. In metabolomics, however, there are still currently various limitations, which, if not addressed, result in abnormally noisy data. Here, we developed a simulation and analysis tool for applying statistical techniques in a training and testing framework, to calculate and correct for technical errors in a dataset and to identify potential outliers. We applied this analysis tool to maize phenolic compounds, including phenylpropanoids and flavonoids, since they play important functions in the interaction of maize with the environment and provide health benefits to humans [34,35,36]. The here-developed tool has numerous applications, such as combining datasets across studies with differing levels of trueQC sample representation, identifying irregular observations in data, and as an experiment planning resource. An advantage of pseudoDrift is that it is written in R and includes an extensive tutorial (File S1), which makes it accessible to all users, including those without extensive programming experience. Since pseudoDrift uses a train–test procedure, a disadvantage might come from users attempting to apply the method to estimate pseudoQC points from small training batches. To offer the greatest flexibility to users, pseudoDrift does not have any batch restrictions, but rather we include a warning to users within the software documentation in R. While the focus of our study was on a targeted LC–MS method applied to samples prepared from a very large number of maize seedlings, the methods described can be broadly applied to other metabolomics datasets, or any temporally variable data prone to technical errors.

## Figures and Tables

**Table 1:** Summary of samples per batch, type of standard included, and whether QC samples were represented or not.

Batch	Num. Samples	External Standard	Internal Standard	QC Samples Represented
B1	165	Yes	No	No
B2	198	Yes	No	No
B3	663	Yes	No	No
B4	1008	Yes	Yes	Yes

## Data Availability

The data underlying this study, including raw data files, intermediate and final peak area matrices are openly available through the CyVerse Data Commons at https://doi.org/10.25739/e1bq-kh07 (accessed on 11 April 2022). The code used for normalizing and applying the pseudoDrift workflow to the maize phenolic LC–MS data is available at https://github.com/jrod55/m_lcms (accessed on 11 April 2022).

## Supplementary Materials

The following supporting information can be downloaded at: https://www.mdpi.com/article/10.3390/metabo12050435/s1, File S1: Tutorial for pseudoDrift and accompanying supplementary figures corresponding to simulated data and analyses conducted with pseudoDrift functions. File S2: Signal drift trends for all compounds retained after data cleaning. In each plot, arbitrary units of area (AUA) are plotted against the injection order. On each page, (A) is the data before applying the signal drift and batch correction with pseudoQCs, and (B) is the plot after applying the correction. Table S1: Targeted multiple reaction monitoring (MRM) method for 33 phenolic compounds investigated in maize seedling (21-day-old) stem tissue. Included for each compound are the name, CAS Registry Number, retention time, polarity mode used during data acquisition, MS/MS (+/−) fragments, and the molecular weight. Additional columns designate the limit of detection (LOD) determined for each sub-batch. Table S2: Parameters solved for ‘pseudo_sdc()’ when applied to the maize seedling (21-day-old) data, which minimize the mean squared error between pseudoQC and trueQC samples when using batch 4 (B4) as the training batch.
