# Supplementary material for: Normalizing and Correcting Variable and Complex LC–MS Metabolomic Data with the R Package pseudoDrift
Source: Metabolites. 2022 May 12;12(5):435. doi: 10.3390/metabo12050435 (PMC9144304; doi:10.3390/metabo12050435)
Supplement: Supplementary file 1 [file metabolites-12-00435-s001.zip › File S1.pdf]

Tutorial for pseudoDrift and accompanying supplementary figures corresponding to simulated data and analyses conducted with pseudoDrift functions.

Introduction

We will demonstrate the functionality of pseudoDrift by data simulation followed by outlier identification then signal drift correction. To get started, make sure the most recent development version from GitHub has been installed. Throughout this document, QC is meant to describe quality control or replicate samples of the same type evaluated within and or between batches in metabolomics studies. These are typically used to identify and correct technical errors or other anomalies which may occur during the data acquisition phase of an experiment.

Throughout this document we make use of the auxiliary R packages loaded below, which include tidyverse,[1] data.table,[2] cowplot,[3] ggpubr,[4] and caret.[5]

Data simulation

To simulate data, an indexed and validated .sdf file is needed to pull the metadata associated with the compound of interest. Here, we use a small .sdf file which is included as part of this package. To construct a validated file from scratch, simply run the commands below setting the paths to your desired .sdf file. The `sdf2index` function is simply a wrapper for ChemmineR R package[6] functions to create an indexed file so that the user does not need to load an enrire .sdf file into memory. The test.sdf file included in our package was obtained from the MoNA online database.

```
# An example sdf2index(sdf_input =
# '/path_to_your/desired.sdf', out_name = 'Index.xls')

# This can run, but not necessary for this vignette
# sdf2index(sdf_input = system.file('extdata', 'test.sdf',
# package = 'pseudoDrift'), out_name = 'Index.xls')
```

The `simulate_data` function takes as input a compound name (which should be present in the .sdf file) or a db ID (also should be present in the .sdf file) and simulates peak matrices using m/z values for the queried compound(s).The number of batches `nbatch`, number of samples per batch `nsamps_per_batch`, and the frequency of QC samples `QC_freq` in each batch can be adjusted by changing the commented lines below. There are additional parameters which allow the user to adjust the variance, scale, and severity of batch and drift effects. Lastly, changing the `seed` will generate a new simulation.

```
sim1 = simulate_data(compound_names = "tricin",
                     xls_file_name = system.file("extdata", "Index.xls",
                                                 package = "pseudoDrift"),
```

```r
                            valid_sdf_file = system.file("extdata", "valid-test.sdf",
                                                          package = "pseudoDrift")
                            # nbatch = 3,
                            # nsamps_per_batch = c(100, 200, 300),
                            # QC_freq = c(25, 25, 25),
                            # seed = 123
                            )
```

The chunk of code above generates simulated data for each instance of 'tricin' found in the .sdf file. Here we are using the default parameters (commented out) which simulate data for three batches, each with one hundred more samples than the previous batch, and with a QC sample included every twenty five samples.

The sim1 object is a nested tibble which contains all metadata available for the compound, along with the simulated data (sim_mat) and four signal drift and batch effect types: 1) monotonic (t1_sim_mat) , 2) batch-to-batch block effect (t2_sim_mat) , 3) random (t3_sim_mat), 4) monotonic coupled with batch-to-batch effect (t4_sim_mat).

Column names of the nested tibble returned by `simulate_data`

```r
names(sim1)
```

```
##  [1] "id"                  "sim_mat"             "t1_sim_mat"
##  [4] "t2_sim_mat"          "t3_sim_mat"          "t4_sim_mat"
##  [7] "name_in_tibble"      "name"                "synonyms"
## [10] "precursor_type"      "spectrum_type"       "precursor_m_z"
## [13] "instrument_type"     "instrument"          "collision_energy"
## [16] "ion_mode"            "inchikey"            "formula"
## [19] "exact_mass"          "mw"                  "contributor"
## [22] "comment"             "num_peaks"           "mass_spectral_peaks"
```

Note that in the tibble above, columns 2:6 are nested tibbles which is where the simulation outputs are stored. We can isolate an individual row using the compound id from in the first column then pull the corresponding simulated data using list indexing syntax. In the code below, we are isolating the simulated data from the first id: FIO00738.

```r
# Simulated data for first id
sim1_sub = sim1 %>%
    filter(id == "FIO00738")

# Simulated, no effects
sim_dat = sim1_sub$sim_mat[[1]]

# Monotonic
mono = sim1_sub$t1_sim_mat[[1]]

# Batch-to-batch
b2b = sim1_sub$t2_sim_mat[[1]]

# Random
rando = sim1_sub$t3_sim_mat[[1]]

# Monotonic and batch-to-batch
mono_b2b = sim1_sub$t4_sim_mat[[1]]
```

Now, to visualize the signal drift trends simulated, highlighting the QC samples we simulated.

```
# Small plotting function
plt_fun = function(x, dat_lab) {
    qc_samps = x %>%
        filter(sample == "QC")
    plt = ggplot(x, aes(batch_index, area)) + geom_point(size = 0.75) +
        geom_point(size = 2, data = qc_samps, aes(color = sample)) +
        geom_line(data = qc_samps, aes(color = sample)) + facet_grid(cols = vars(batch),
        scales = "free") + labs(title = paste0(dat_lab), y = "area_simulated") +
        scale_y_continuous(limits = c(0, 80000))
    return(plt)
}
```

```
p1 = plt_fun(sim_dat, "Simulated")
p2 = plt_fun(mono, "Monotonic")
p3 = plt_fun(b2b, "Batch-to-batch")
p4 = plt_fun(rando, "Random")
p5 = plt_fun(mono_b2b, "Monotonic and batch-to-batch")
legend = get_legend(p1)

p_all = plot_grid(p1 + rremove("legend"), p2 + rremove("legend"),
    p3 + rremove("legend"), p4 + rremove("legend"), p5 + rremove("legend"),
    labels = "AUTO", ncol = 1)

p_all = plot_grid(p_all, legend, ncol = 2, rel_widths = c(1,
    0.1))

p_all
```

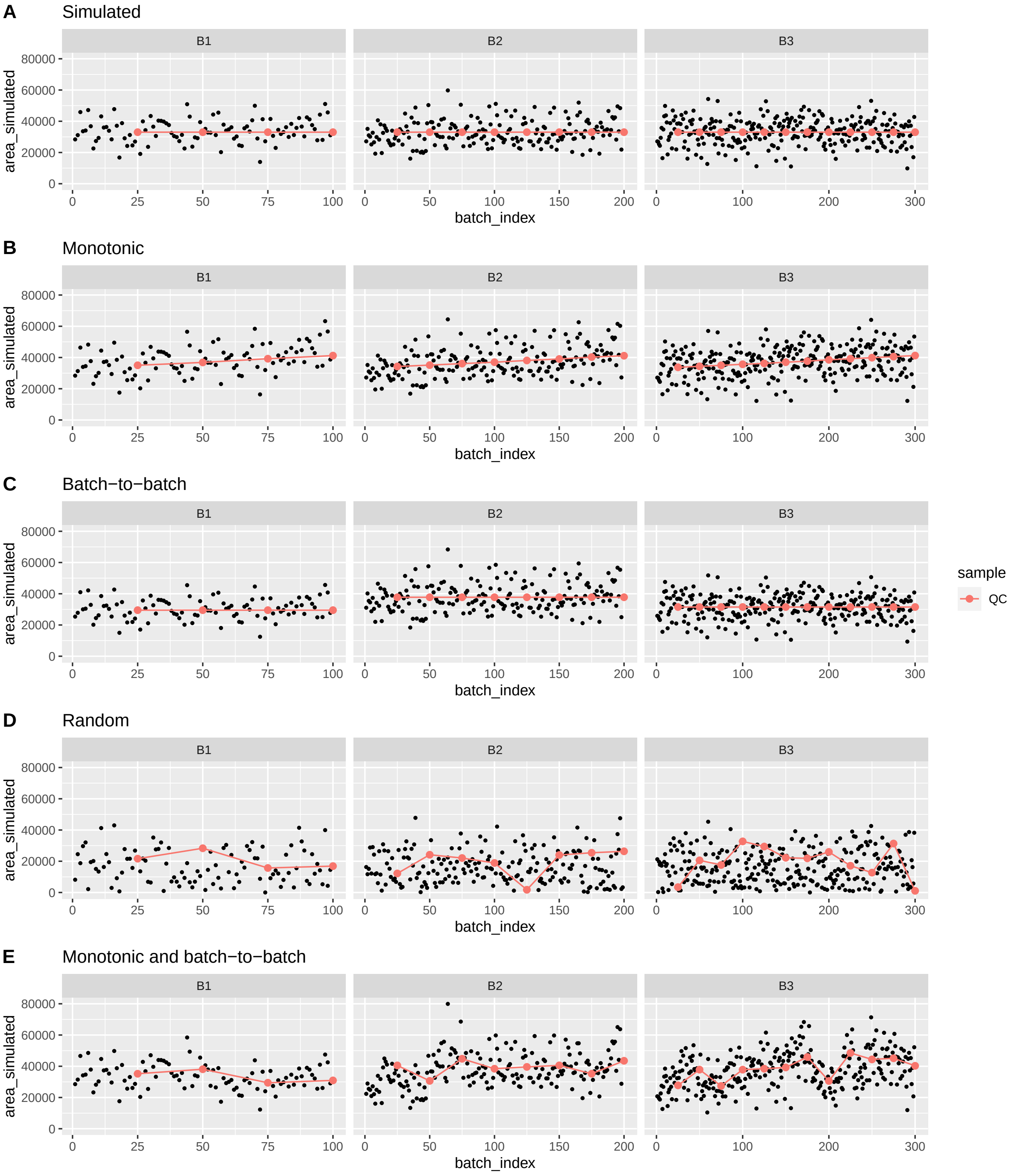

Figure S1: Scatterplot visualization of the simulated dataset (A), and the different batch effect and signal drift types, including type 1 monotonic (B), type2 batch-to-batch (C), tpye 3 random (D), and type4 monotonic plus batch-to-batch (E). Each plot also includes a line plot connecting the QC samples included at the desired frequency during the simulation.

For the remainder of this document, the focus will be on the monotonic + batch-to-batch simulated data. Primarily because this is what is most typically observed in real data sets.

Pairwise outlier identification

It is a challenging task to identify and remove outliers from any set of data, and having limited replication can further complicate this task. Here we introduce an outlier identification method `pw_outlier` which compares within sample pairwise differences (among individual sample replicates) to the distribution of all differences among samples. To illustrate how this procedure works, we will analyze the simulated data with the `pw_outlier` function.

```
# Assign replicates to simulated data.  Note no technical
# replicates are included here, we are assuming only
# biological replicates.

n_reps = 3
qcs = mono_b2b %>%
    filter(sample == "QC")
tmp = mono_b2b %>%
    filter(!sample == "QC") %>%
    mutate(n_samps = n()/all_of(n_reps))
dat_rep = rep(1:n_reps, unique(tmp$n_samps))
dat_sam = paste0("S", rep(1:unique(tmp$n_samps), each = n_reps))

# The Monotonic + batch-to-batch simulated data with
# replicateds assigned
mono_b2b_WR = tmp %>%
    mutate(rep = all_of(dat_rep), sample = all_of(dat_sam), rep_tech = 1) %>%
    bind_rows(., qcs) %>%
    arrange(experiment_index)

pw_out = pw_outlier(df = mono_b2b_WR, return_plot = TRUE, samps_exclude = "QC")
```

```
##
##  --------------
##  Done!
##  --------------
```

```
pw_out$batch_plots
```

```
## [[1]]
```

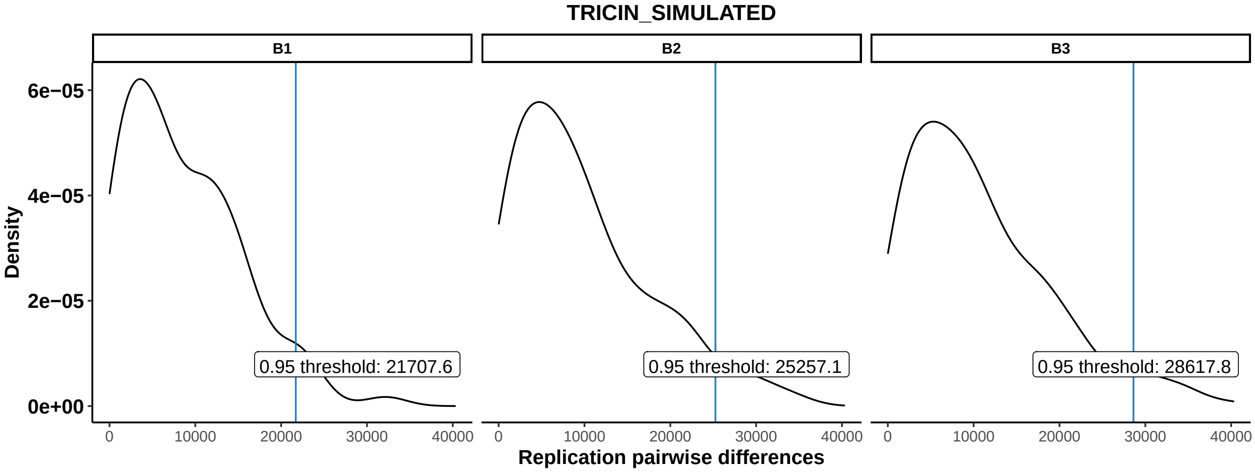

Figure S2: Diagnostic plots returned by `pw_outlier`. The density curve plotted for each batch represents all pairwise differences within each triplicate set in the simulated data. The blue vertical lines are the 0.95% threshold value calculated for each batch.

To clarify how pairwise differences are computed, consider the following example: Sample S1 with reps 1, 2, 3 would have pairwise differences computed as |1-2|, |1-3|, |2-3|. The density curves plotted above in Figure S2 show that most differences are small, as expected, which results in a positively skewed distribution.

```
# Make a plot of the data removed by pw_outlier
df_rm = pw_out$df_rm %>%
    mutate(sample = "pw_outlier")

# Base plot for making comparisons
plt_base = plt_fun(pw_out$df_cleaned, "")

## Outliers by pw_outlier
pw_plt = plt_base + geom_point(data = df_rm, size = 1.25, aes(color = sample)) +
    scale_color_manual(values = c("#f8766D", "blue"), limits = c("QC",
        "pw_outlier")) + theme(legend.title = element_blank())

## 1.5 IQR and iterative Grubs test outlier detection
## function
library(outliers)
outlier_conventional = function(x) {
    grubs_outliers = NULL
    test = x
    grubbs.result = grubbs.test(test)
    pv = grubbs.result$p.value
    while (pv < 0.05) {
        grubs_outliers = c(grubs_outliers, as.numeric(strsplit(grubbs.result$alternative,
            " ")[[1]][3]))
        test = x[!x %in% grubs_outliers]
        grubbs.result = grubbs.test(test)
        pv = grubbs.result$p.value
    }
    iqr_outliers = boxplot.stats(x)$out
    return(data.frame(grubs_outliers = (x %in% grubs_outliers),
        iqr_outliers = (x %in% iqr_outliers)))
```

```r
}

## Conventional outlier tests
conv_out_tests = pw_out$df %>%
    group_by(batch) %>%
    nest() %>%
    mutate(my_rosner = map(data, ~outlier_conventional(.x$area))) %>%
    unnest(cols = c(data, my_rosner))

## Outlier by IQR
iqr_dat = conv_out_tests %>%
    filter(iqr_outliers == "TRUE") %>%
    mutate(sample = "IQR_outlier", sample = factor(sample, levels = c("QC",
        "IQR_outlier")))

iqr_plt = plt_base + geom_point(data = iqr_dat, size = 1.25,
    aes(color = sample)) + scale_color_manual(values = c("#f8766D",
    "purple"), limits = c("QC", "IQR_outlier")) + theme(legend.title = element_blank())

## Outlier by grubbs
grubbs_dat = conv_out_tests %>%
    filter(grubs_outliers == "TRUE") %>%
    mutate(sample = "Grubbs_outlier", sample = factor(sample,
        levels = c("Grubbs_outlier", "QC")))

grubbs_plt = plt_base + geom_point(data = grubbs_dat, size = 1.25,
    aes(color = sample)) + scale_color_manual(values = c("#f8766D",
    "red"), limits = c("QC", "Grubbs_outlier")) + theme(legend.title = element_blank())

## Join the plots
comp_plt = plot_grid(pw_plt, iqr_plt, grubbs_plt, ncol = 1, align = "hv",
    axis = c("tblr"), labels = "AUTO")

comp_plt
```

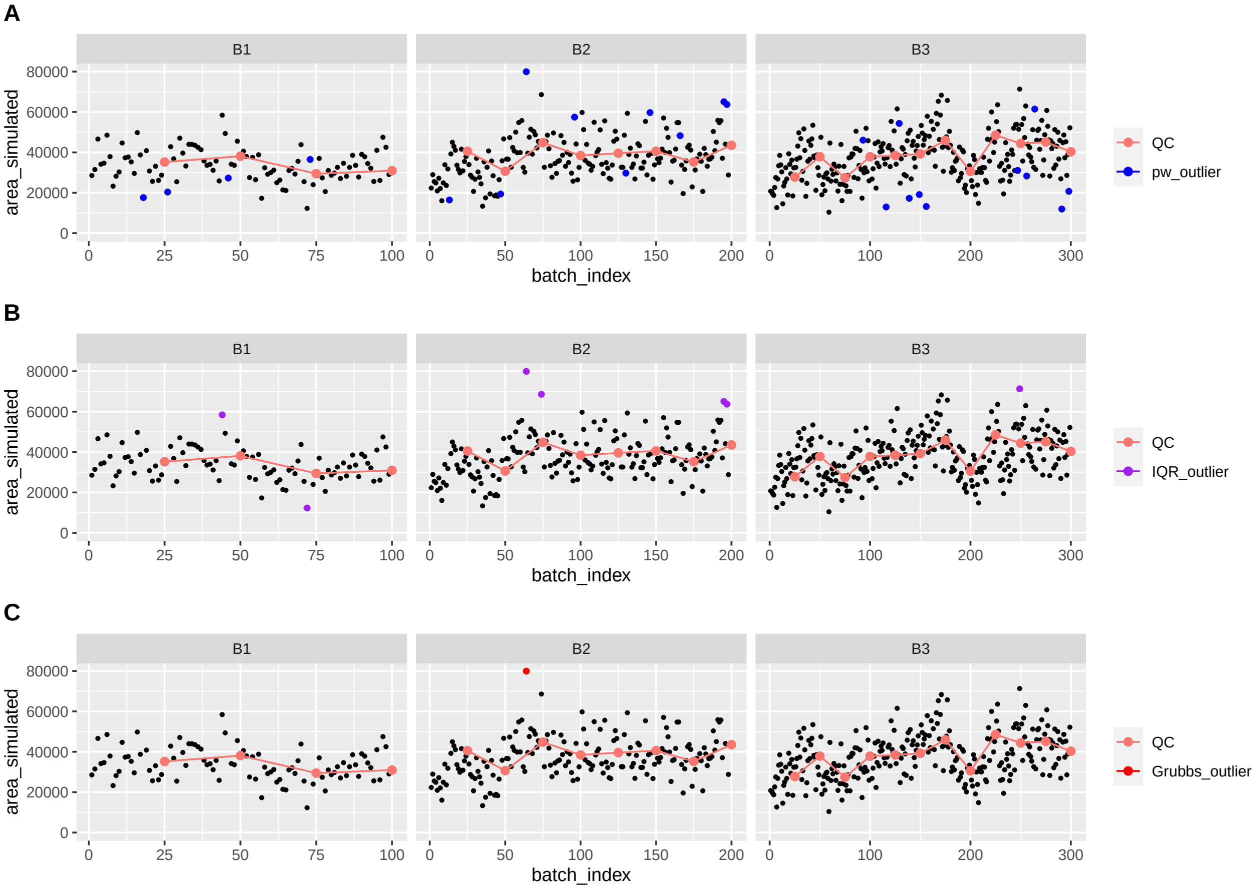

Figure S3: Comparison of three different outlier detection methods: (A) pw_oultier, (B) 1.5*Inter-Quartile-Range (IQR) and (C) Grubbs outlier test.

Signal drift correction

Suppose that QC samples were included in batch 3 alone. This would present issues for QC-based correction methods which adjust the data relying on the presence of QCs across all batches. We introduce a method `pseudo_sdc` to estimate 'pseudoQC' samples from all non-QC samples in a training batch.

The training batch contains QC samples and is used to find the optimal set of four parameters (`test.breaks`, `test.window`, `test.index`, `quantile_range`) which are used to estimate pseudoQCs and minimize the `criteria` (set by the user) between pseudoQCs and trueQCs.

Internally, the `psuedo_sdc` function fits all possible regression splines with pseudoQC samples calculated as a function of each combination of parameters given as inputs. The regression splines are fit using the training batch data to optimize the parameters then the same set of parameters is applied to the test data, which does not have trueQC samples.

```
# Use the pw_outlier cleaned data
mono_b2b_cleaned = pw_out$df_cleaned

# A good starting point for parameters to test
```

```r
train.batch = "B3"
df_param = mono_b2b_cleaned %>%
    filter(!sample == "QC") %>%
    group_by(batch) %>%
    summarise(samps = n_distinct(name))

# Sample size of smallest batch
s_perBatch = min(df_param$samps)

# Test breaks in smallest batch, using the number of QC
# samples simulated there
mnqc = 25
t.b = round(s_perBatch/mnqc) + 1

# Proportional in larger training set
df_param = df_param %>%
    mutate(bre = round(samps * all_of(t.b)/all_of(s_perBatch)))
t.b.train = df_param %>%
    filter(batch == all_of(train.batch)) %>%
    pull(bre)
t.b.train = seq(t.b.train - 3, t.b.train + 3, 1)

# Test window for median smoothing. Should be a vector of
# odd numbers
w.n.max = round(min(s_perBatch/t.b))/2
w.n = 2 * floor(w.n.max/2) + 1
if (w.n > w.n.max) {
    w.n = w.n - 2
}
w.n = seq(1, w.n, 2)

# Test index offset. Larger values can be tested with
# larger data sets ti.max = round(s_perBatch*0.15) t.i =
# seq(0,ti.max,1)

## Test breaking up the training batch into 10 to 12
## equally sized sections test.breaks = t.b.train

## Test taking the median every 1 (reduces to the mean per
## test.break), to 9 samples. This sequence of numbers
## should be all odd.  test.window = w.n

## Test offsetting the injection order of pseudoQC samples
## estimated test.index = t.i

## This can be one of: 'RSD' (relative standard deviation
## using standard deviation and mean) 'RSD_robust'
## (relative standard deviation using median absolute
## deviation and median) 'MSE' (mean squared error), or
## 'TSS' (total sum of squares).  This is the criteria to
## be minimized.  criteria = 'MSE'

## Number of cores to use if your machine has the ability
```

```r
## to multi-thread processes.  n.cores = 12

## Quantile values (above/below increment) of the data
## surrounding QCs to use to estimate pseudoQCs.
## quantile.increment = 0.05

# To reduce computing time, we are going to use values
# previously obtained by running the exhaustive set of
# 16,800 tests with the inputs commented out above.
test.breaks = 12
test.window = 7
test.index = 5
criteria = "MSE"
quantile.increment = 0.05
n.cores = 1

sdc_out = pseudo_sdc(df = mono_b2b_cleaned, n.cores = n.cores,
    train.batch = train.batch, test.breaks = test.breaks, test.window = test.window,
    test.index = test.index, criteria = criteria, qc.label = "QC",
    min.qc = min(test.breaks), quantile.increment = quantile.increment)
```

```
## [1] "Running...testing 32 combinations of input parameters"
## [1] "The best fit for TRICIN_SIMULATED in training batch B3 is:"
## [1] "With median smoothing every 7 samples to generate pseudo-pools."
## [1] "Index (injection order) offset by 5, and splitting the batch into 12 sections."
## [1] "Using peak area values between quantiles 0.1 and 0.95"
##
##  --------------
##  Done!
##  --------------
```

```r
# Note the optimal parameters using the non-selected
# criteria are also returned. Here, recall we only ran with
# the parameters optimized for MSE therefore the other
# values in the table below may not be the true minima for
# the respective criteria. To see the full table uncomment
# the line below

# sdc_out$criteria_table
```

```r
# A slightly larger plotting function
plt_fun1 = function(x, train.batch) {
    metab = unique(x$df$compound)
    x1 = x$df_pseudoQC %>%
        group_by(batch) %>%
        mutate(index = 1:n(), class = factor(class, levels = c("QC",
            "Pseudo_QC", "Sample")))

    qcs1 = x1 %>%
        filter(class %in% c("QC", "Pseudo_QC")) %>%
        mutate(sample = class)

    plt1 = ggplot(x1, aes(index, area)) + geom_point() + geom_point(data = qcs1,
```

```
        aes(color = sample)) + geom_line(data = qcs1, aes(color = sample)) +
        facet_grid(cols = vars(batch), scales = "free") + labs(title = paste0(metab,
        " raw data using ", train.batch, " data to train pseudo-QC"))

    legend = get_legend(plt1)

    x2 = x$df_pseudoQC_corrected %>%
        group_by(batch) %>%
        mutate(index = 1:n(), class = factor(class, levels = c("QC",
            "Pseudo_QC", "Sample")))

    qcs2 = x2 %>%
        filter(class %in% c("QC", "Pseudo_QC")) %>%
        mutate(sample = class)

    plt2 = ggplot(x2, aes(index, area_corrected)) + geom_point() +
        geom_point(data = qcs2, aes(color = sample)) + geom_line(data = qcs2,
        aes(color = sample)) + facet_grid(cols = vars(batch),
        scales = "free") + labs(title = paste0(metab, " pseudo-QC corrected data"))

    cplt = plot_grid(plt1, plt2, ncol = 1, labels = "AUTO")
    return(cplt)
}

plt_fun1(sdc_out, train.batch)
```

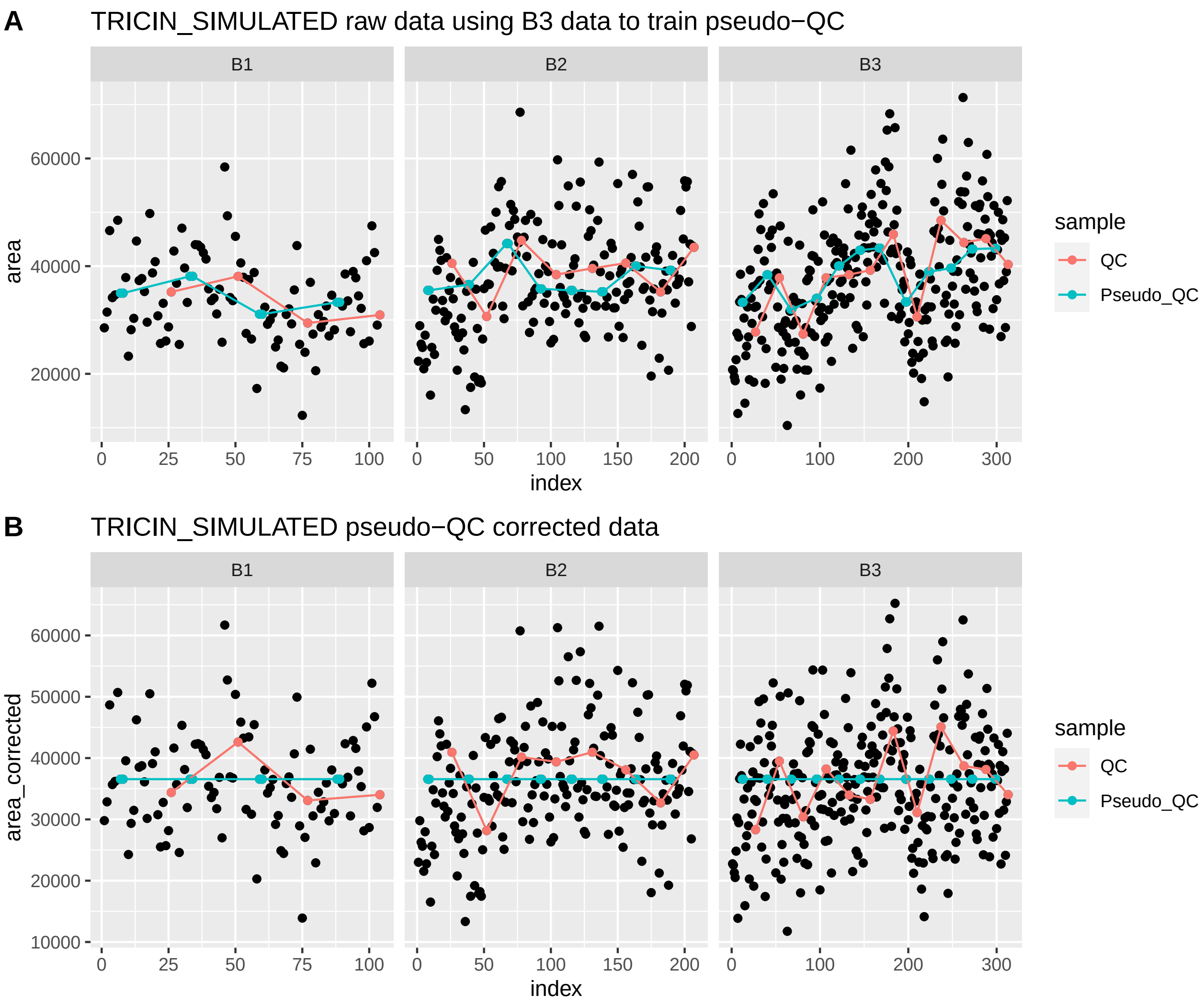

Figure S4: Type 4 simulated data with trueQC and pseudoQC samples highlighted before correction (A) and after signal drift and batch correction using pseudoQC samples to do correction (B).

```
## RSD-QC before correction
rsd_qc_before = sdc_out$df %>%
    filter(sample == "QC") %>%
    summarise(rsd_before = mad(area, na.rm = TRUE)/abs(median(area,
        na.rm = TRUE)))

## After correction with pseudoQC
rsd_qc_after = sdc_out$df_pseudoQC_corrected %>%
    filter(sample == "QC") %>%
    summarise(rsd_after = mad(area_corrected, na.rm = TRUE)/abs(median(area_corrected,
        na.rm = TRUE)))

cbind(rsd_qc_before, rsd_qc_after)
```

```
##   rsd_before rsd_after
## 1  0.1601129 0.1593009
```

Comparing correction methods with the originally simulated data

In the previous section we used RSD-QC to assess the performance of our correction method implemented in `pseudo_sdc`. Since we know the true value of the originally simulated data without any effects at all, we can test the performance of our method considering all data points. Then, compare this correction with doing a trueQC based correction using the pmp R package.[7]

```
## Join corrected and simulated data
cor_dat = sdc_out$df_pseudoQC_corrected %>%
    select(name, area_corrected, class) %>%
    filter(!class %in% c("QC")) %>%
    left_join(., sim_dat) %>%
    drop_na(area)
```

```
## Joining, by = "name"
```

```
## Fit a simple linear mode and perform 10-fold cross
## validation
set.seed(123)
fc = trainControl(method = "cv", number = 10)
fit = train(area ~ area_corrected, data = cor_dat, method = "lm",
    trControl = fc)

cor_plt = cor_dat %>%
    ggplot(., aes(area_corrected, area)) + geom_point(alpha = 0.5,
    aes(color = batch)) + geom_smooth(method = "lm", color = "black",
    se = TRUE) + geom_text(aes(label = paste0("RMSE: ", signif(fit$results$RMSE,
    4)), y = 1000, x = 55000), show.legend = FALSE, color = "black") +
    geom_text(aes(label = paste0("Rsq: ", signif(fit$results$Rsquared,
        4)), y = 5000, x = 55000), show.legend = FALSE, color = "black") +
    labs(x = "area_corrected_with_pseudoQC", y = "original_area_simulated_no_effects",
        title = "pseudoQC corrected")

## Now perform the correction using true QC samples then
## making the same comparisons.
m_qcrsc = function(x, y) {
    x = x %>%
        mutate(class = ifelse(sample %in% all_of(y), "QC", "Sample"))
    qc_samps = x %>%
        filter(class == "QC")
    ## pmp seems to require at least two QC in tandem
    x = bind_rows(x, qc_samps) %>%
        arrange(experiment_index)
    tmp_names = x$name
    x = x %>%
        mutate(name = paste0(name, row_number()))
    t_meta = colnames(x)
    t_meta = t_meta[!t_meta %in% c("name", "compound")]
    tqc = x %>%
        pivot_wider(id_cols = !all_of(t_meta), names_from = name,
            values_from = area)
    t_rn = tqc$compound
    tqc = as.matrix(tqc[, -1])
```

```
    rownames(tqc) = t_rn
    mc = pmp::QCRSC(df = tqc, minQC = 4, order = seq_along(x$experiment_index),
        batch = x$batch, classes = x$class, qc_label = paste0(y))
    z = x %>%
        mutate(area_corrected = mc[1, ], .before = area)
    z$name = tmp_names
    return(z)
}

## Run the function then conduct the same diagnostics tests
## done with pseudoQC above
true_QC = m_qcrsc(x = mono_b2b_cleaned, y = "QC")
```

```
## The number of NA and <= 0 values in peaksData before QC-RSC: 0
```

```
cor_dat_true_QC = true_QC %>%
    select(name, area_corrected, class) %>%
    filter(!class %in% c("QC")) %>%
    left_join(., sim_dat) %>%
    drop_na(area)
```

```
## Joining, by = "name"
```

```
fit_true_QC = train(area ~ area_corrected, data = cor_dat_true_QC,
    method = "lm", trControl = fc)

cor_plt_true_QC = cor_dat_true_QC %>%
    ggplot(., aes(area_corrected, area)) + geom_point(alpha = 0.5,
    aes(color = batch)) + geom_smooth(method = "lm", color = "black",
    se = TRUE) + geom_text(aes(label = paste0("RMSE: ", signif(fit_true_QC$results$RMSE,
    4)), y = 1000, x = 55000), show.legend = FALSE, color = "black") +
    geom_text(aes(label = paste0("Rsq: ", signif(fit_true_QC$results$Rsquared,
        4)), y = 5000, x = 55000), show.legend = FALSE, color = "black") +
    labs(x = "area_corrected_with_true_QC", y = "", title = "trueQC corrected")


## Combine the plots to show the comparison
legend = get_legend(cor_plt)
```

```
## `geom_smooth()` using formula 'y ~ x'
```

```
tmp = plot_grid(cor_plt + rremove("legend"), cor_plt_true_QC +
    rremove("legend"), labels = "AUTO", nrow = 1)
```

```
## `geom_smooth()` using formula 'y ~ x'
## `geom_smooth()` using formula 'y ~ x'
```

```
method_comparison = plot_grid(tmp, legend, rel_widths = c(1,
    0.1))

method_comparison
```

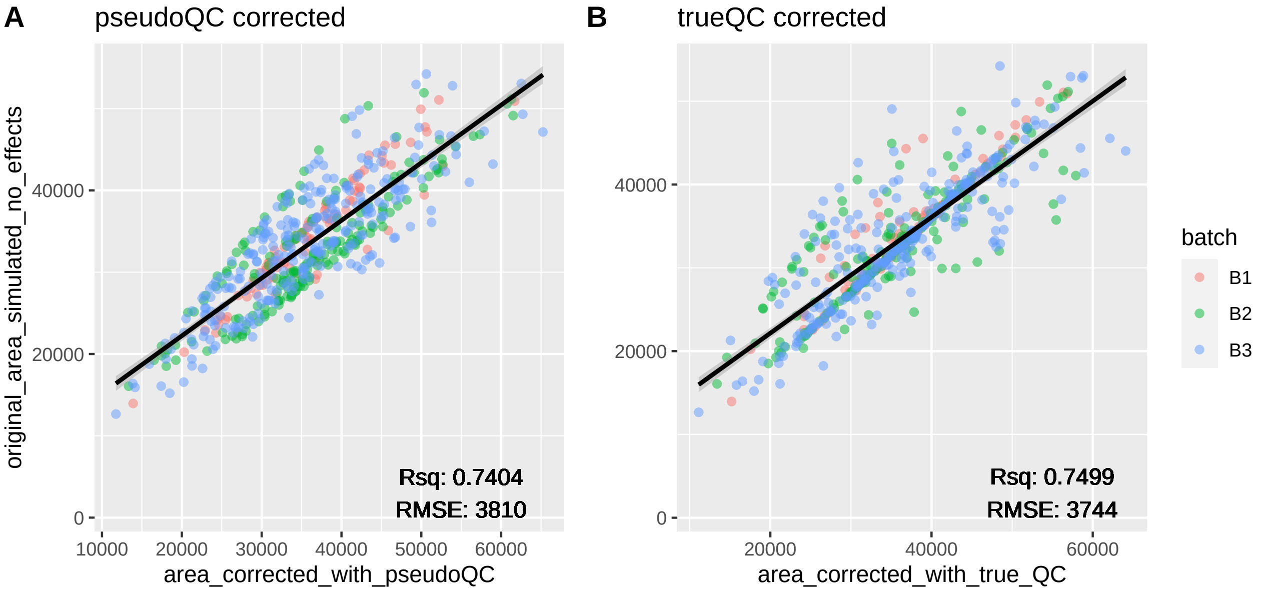

Figure S5: Comparison between pseudoQC (A) and trueQC (B) signal drift and batch effect correction methods with simulated data.

Final remarks

In this document, we've presented the main functionality of pseudoDrift and have provided evidence supporting the ability for pseudoDrift to effectively reduce signal drift and batch effects. Additionally, our package offers a relatively easy way for anyone familiar with basic R programming to learn and or teach concepts related to technical issues in metabolomics data sets.

Session information

```
sessionInfo()
```

```
## R version 4.1.1 (2021-08-10)
## Platform: x86_64-pc-linux-gnu (64-bit)
## Running under: Ubuntu 18.04.5 LTS
##
## Matrix products: default
## BLAS:   /usr/lib/x86_64-linux-gnu/openblas/libblas.so.3
## LAPACK: /usr/lib/x86_64-linux-gnu/libopenblasp-r0.2.20.so
##
## locale:
##  [1] LC_CTYPE=en_US.UTF-8       LC_NUMERIC=C
##  [3] LC_TIME=en_US.UTF-8        LC_COLLATE=en_US.UTF-8
##  [5] LC_MONETARY=en_US.UTF-8    LC_MESSAGES=en_US.UTF-8
##  [7] LC_PAPER=en_US.UTF-8       LC_NAME=C
##  [9] LC_ADDRESS=C               LC_TELEPHONE=C
```

```
## [11] LC_MEASUREMENT=en_US.UTF-8 LC_IDENTIFICATION=C
##
## attached base packages:
## [1] stats     graphics  grDevices utils     datasets  methods   base
##
## other attached packages:
##  [1] outliers_0.14     caret_6.0-88      lattice_0.20-44   ggpubr_0.4.0
##  [5] cowplot_1.1.1     data.table_1.14.0 pseudoDrift_1.0.0 forcats_0.5.1
##  [9] stringr_1.4.0     dplyr_1.0.7       purrr_0.3.4       readr_2.0.1
## [13] tidyr_1.1.4       tibble_3.1.6      ggplot2_3.3.5     tidyverse_1.3.1
##
## loaded via a namespace (and not attached):
##   [1] readxl_1.3.1              backports_1.2.1
##   [3] plyr_1.8.6                splines_4.1.1
##   [5] listenv_0.8.0             usethis_2.0.1
##   [7] ChemmineR_3.44.0          GenomeInfoDb_1.28.2
##   [9] digest_0.6.29             foreach_1.5.1
##  [11] htmltools_0.5.2           fansi_1.0.2
##  [13] magrittr_2.0.2            memoise_2.0.0
##  [15] tzdb_0.1.2                openxlsx_4.2.4
##  [17] remotes_2.4.0             globals_0.14.0
##  [19] recipes_0.1.16            gower_0.2.2
##  [21] modelr_0.1.8              matrixStats_0.60.1
##  [23] vroom_1.5.4               prettyunits_1.1.1
##  [25] colorspace_2.0-2          rvest_1.0.1
##  [27] haven_2.4.3               xfun_0.25
##  [29] callr_3.7.0               crayon_1.4.2
##  [31] RCurl_1.98-1.4            jsonlite_1.7.2
##  [33] missForest_1.4            impute_1.66.0
##  [35] survival_3.2-13           zoo_1.8-9
##  [37] iterators_1.0.13          glue_1.6.1
##  [39] gtable_0.3.0              ipred_0.9-11
##  [41] zlibbioc_1.38.0           XVector_0.32.0
##  [43] DelayedArray_0.18.0       car_3.0-11
##  [45] pkgbuild_1.2.0            future.apply_1.8.1
##  [47] BiocGenerics_0.38.0       abind_1.4-5
##  [49] scales_1.1.1              DBI_1.1.1
##  [51] rstatix_0.7.0             Rcpp_1.0.8
##  [53] foreign_0.8-81            bit_4.0.4
##  [55] lava_1.6.10               prodlim_2019.11.13
##  [57] stats4_4.1.1              rsvg_2.1.2
##  [59] DT_0.19                   htmlwidgets_1.5.3
##  [61] httr_1.4.2                ellipsis_0.3.2
##  [63] farver_2.1.0              pkgconfig_2.0.3
##  [65] nnet_7.3-16               dbplyr_2.1.1
##  [67] janitor_2.1.0             utf8_1.2.2
##  [69] labeling_0.4.2            tidyselect_1.1.1
##  [71] rlang_1.0.0               reshape2_1.4.4
##  [73] munsell_0.5.0             cellranger_1.1.0
##  [75] tools_4.1.1               cachem_1.0.6
##  [77] cli_3.1.1                 generics_0.1.1
##  [79] devtools_2.4.2            broom_0.7.9
##  [81] evaluate_0.14             fastmap_1.1.0
##  [83] yaml_2.2.1                ModelMetrics_1.2.2.2
```

```
##  [85] processx_3.5.2              knitr_1.33
##  [87] bit64_4.0.5                 fs_1.5.0
##  [89] zip_2.2.0                   randomForest_4.6-14
##  [91] future_1.22.1               nlme_3.1-152
##  [93] pmp_1.4.0                   formatR_1.11
##  [95] xml2_1.3.2                  compiler_4.1.1
##  [97] rstudioapi_0.13             curl_4.3.2
##  [99] png_0.1-7                   testthat_3.0.4
## [101] ggsignif_0.6.2              reprex_2.0.1
## [103] stringi_1.7.4               ps_1.6.0
## [105] desc_1.3.0                  Matrix_1.3-4
## [107] vctrs_0.3.8                 pillar_1.6.5
## [109] lifecycle_1.0.1             bitops_1.0-7
## [111] GenomicRanges_1.44.0        R6_2.5.1
## [113] pcaMethods_1.84.0           gridExtra_2.3
## [115] rio_0.5.27                  parallelly_1.27.0
## [117] IRanges_2.26.0              sessioninfo_1.1.1
## [119] codetools_0.2-18            MASS_7.3-54
## [121] assertthat_0.2.1            pkgload_1.2.1
## [123] SummarizedExperiment_1.22.0 rprojroot_2.0.2
## [125] rjson_0.2.20                withr_2.4.3
## [127] S4Vectors_0.30.0            GenomeInfoDbData_1.2.6
## [129] mgcv_1.8-36                 parallel_4.1.1
## [131] hms_1.1.1                   rpart_4.1-15
## [133] timeDate_3043.102           grid_4.1.1
## [135] class_7.3-19                snakecase_0.11.0
## [137] rmarkdown_2.10              MatrixGenerics_1.4.3
## [139] carData_3.0-4               pROC_1.18.0
## [141] itertools_0.1-3             Biobase_2.52.0
## [143] lubridate_1.7.10            base64enc_0.1-3
```


(1) Wickham, H.; Averick, M.; Bryan, J.; Chang, W.; McGowan, L. D.; François, R.; Grolemund, G.; Hayes, A.; Henry, L.; Hester, J.; Kuhn, M.; Pedersen, T. L.; Miller, E.; Bache, S. M.; Müller, K.; Ooms, J.; Robinson, D.; Seidel, D. P.; Spinu, V.; Takahashi, K.; Vaughan, D.; Wilke, C.; Woo, K.; Yutani, H. Welcome to the tidyverse. *Journal of Open Source Software* **2019**, *4* (43), 1686. https://doi.org/10.21105/joss.01686.

(2) Dowle, M.; Srinivasan, A. *Data.table: Extension of 'Data.frame'*; 2021.

(3) Wilke, C. O. *Cowplot: Streamlined Plot Theme and Plot Annotations for 'Ggplot2'*; 2020.

(4) Kassambara, A. *Ggpubr: 'Ggplot2' Based Publication Ready Plots*; 2020.

(5) Kuhn, M. *Caret: Classification and Regression Training*; 2021.

(6) Cao, Y. E.; Horan, K.; Backman, T.; Girke, T. *ChemmineR: Cheminformatics Toolkit for r*; 2021.

(7) Jankevics, A.; Lloyd, G. R.; Weber, R. J. M. *Pmp: Peak Matrix Processing and Signal Batch Correction for Metabolomics Datasets*; 2021.
