# Supplementary material for: Normalizing and Correcting Variable and Complex LC–MS Metabolomic Data with the R Package pseudoDrift
Source: Metabolites. 2022 May 12;12(5):435. doi: 10.3390/metabo12050435 (PMC9144304; doi:10.3390/metabo12050435)
Supplement: Supplementary file 1 [file metabolites-12-00435-s001.zip › File S2.pdf]

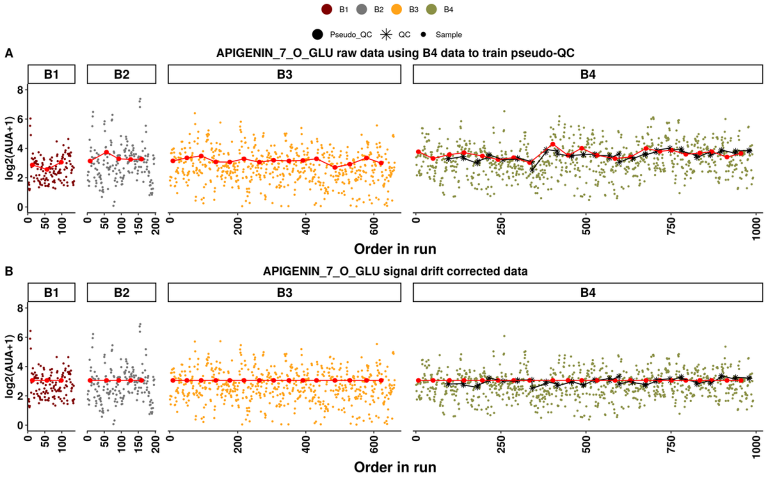

B1
B2
B3
B4
Pseudo_QC
QC
Sample
A
APIGENIN_7_O_GLU raw data using B4 data to train pseudo-QC
log2(AUA+1)
Order in run
B
APIGENIN_7_O_GLU signal drift corrected data
log2(AUA+1)
Order in run

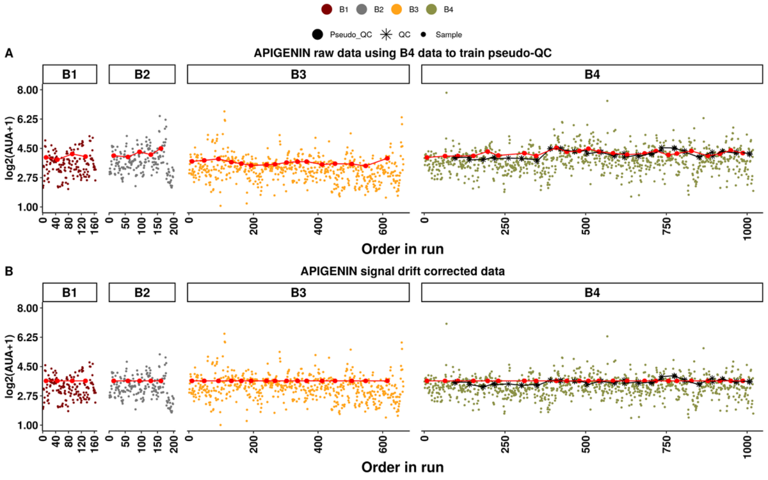

B1
B2
B3
B4
Pseudo_QC
QC
Sample
A
APIGENIN raw data using B4 data to train pseudo-QC
B
APIGENIN signal drift corrected data
log2(AUA+1)
Order in run

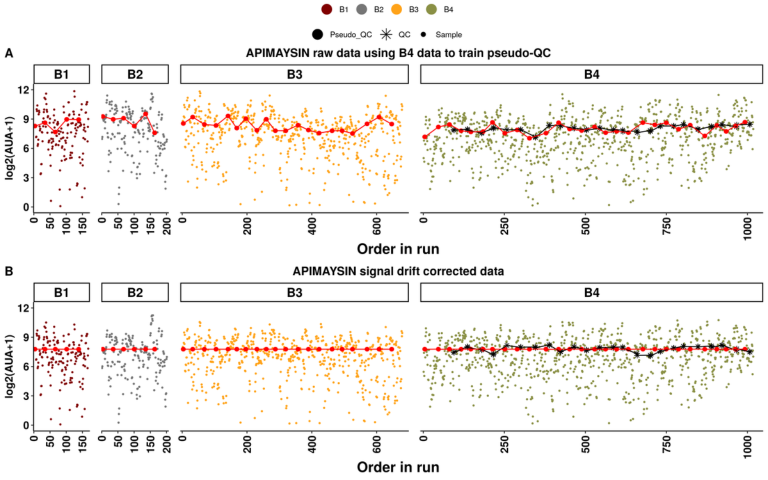

B1
B2
B3
B4
Pseudo_QC
QC
Sample
A
APIMAYSIN raw data using B4 data to train pseudo-QC
log2(AUA+1)
Order in run
B
APIMAYSIN signal drift corrected data
log2(AUA+1)
Order in run

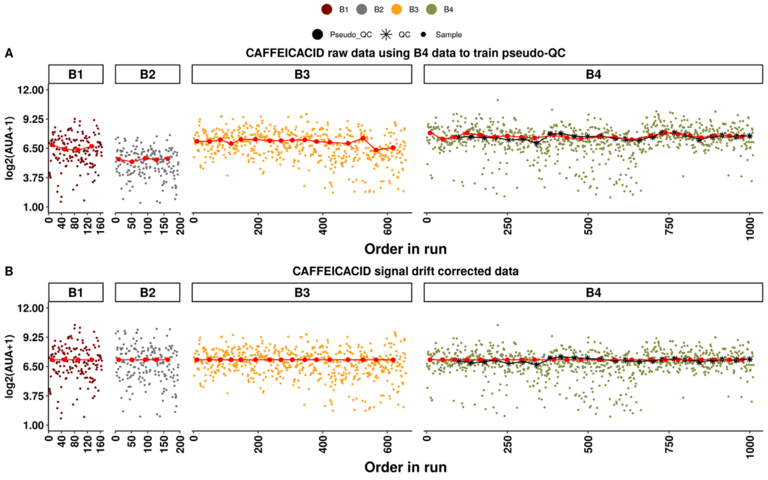
B1
B2
B3
B4
Pseudo_QC
QC
Sample
A
CAFFEICACID raw data using B4 data to train pseudo-QC
log2(AUA+1)
12.00
9.25
6.50
3.75
1.00
Order in run
B
CAFFEICACID signal drift corrected data
0
40
80
120
160
50
100
150
200
400
600
250
500
750
1000

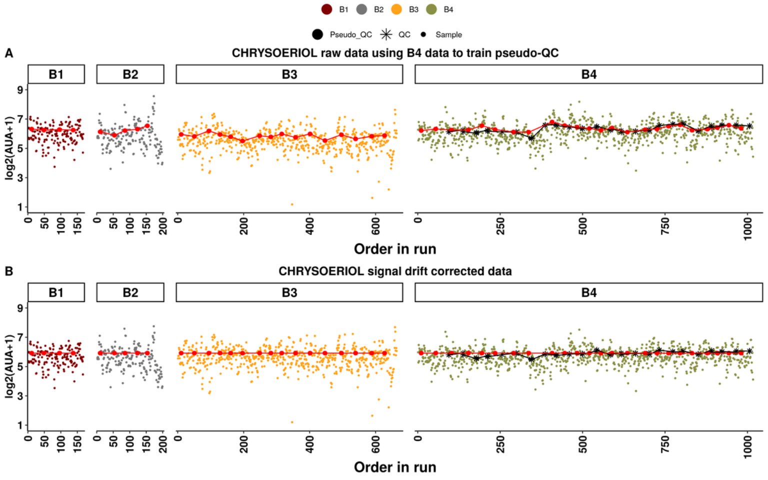

B1
B2
B3
B4
Pseudo_QC
QC
Sample
A
CHRYSOERIOL raw data using B4 data to train pseudo-QC
log2(AUA+1)
Order in run
B
CHRYSOERIOL signal drift corrected data
log2(AUA+1)
Order in run

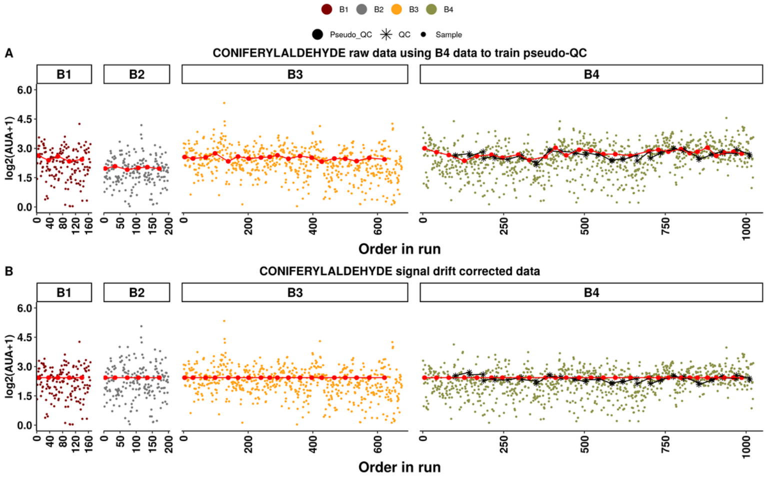
B1
B2
B3
B4
Pseudo_QC
QC
Sample
A
CONIFERYLALDEHYDE raw data using B4 data to train pseudo-QC
B1
B2
B3
B4
log2(AUA+1)
6.0
4.5
3.0
1.5
0.0
0
40
80
120
160
0
50
100
150
200
0
200
400
600
0
250
500
750
1000
Order in run
B
CONIFERYLALDEHYDE signal drift corrected data
B1
B2
B3
B4
log2(AUA+1)
6.0
4.5
3.0
1.5
0.0
0
40
80
120
160
0
50
100
150
200
0
200
400
600
0
250
500
750
1000
Order in run

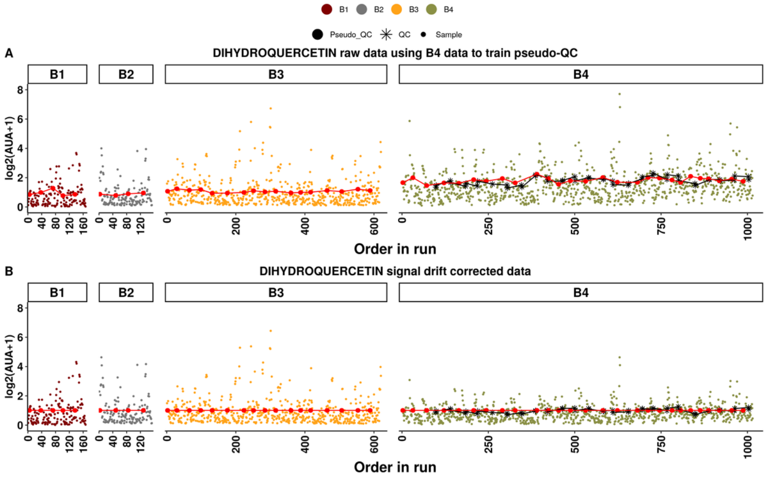
B1 B2 B3 B4
Pseudo_QC QC Sample
A
DIHYDROQUERCETIN raw data using B4 data to train pseudo-QC
B1
B2
B3
B4
log2(AUA+1)
Order in run
B
DIHYDROQUERCETIN signal drift corrected data
B1
B2
B3
B4
log2(AUA+1)
Order in run

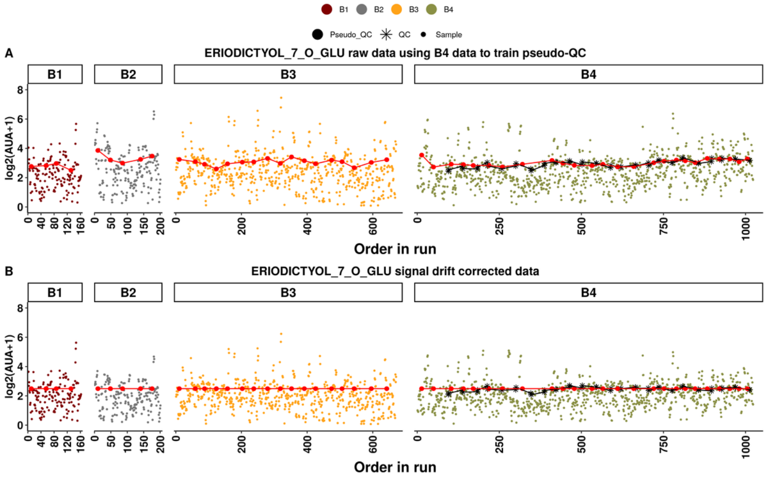

B1
B2
B3
B4
Pseudo_QC
QC
Sample
A
ERIODICTYOL_7_O_GLU raw data using B4 data to train pseudo-QC
B1
B2
B3
B4
log2(AUA+1)
0
2
4
6
8
0
40
80
120
160
0
50
100
150
200
0
200
400
600
0
250
500
750
1000
Order in run
B
ERIODICTYOL_7_O_GLU signal drift corrected data
B1
B2
B3
B4
log2(AUA+1)
0
2
4
6
8
0
40
80
120
160
0
50
100
150
200
0
200
400
600
0
250
500
750
1000
Order in run

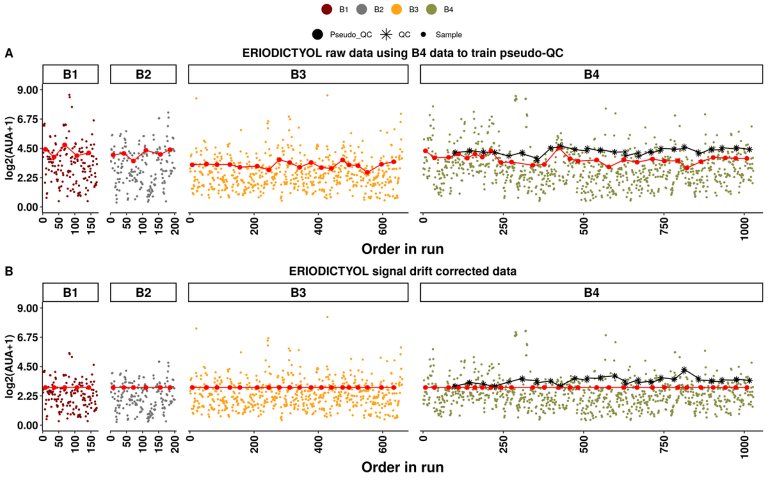

B1
B2
B3
B4
Pseudo_QC
QC
Sample
A
ERIODICTYOL raw data using B4 data to train pseudo-QC
B1
B2
B3
B4
9.00
6.75
4.50
2.25
0.00
log2(AUA+1)
0
50
100
150
200
400
600
250
500
750
1000
Order in run
B
ERIODICTYOL signal drift corrected data
B1
B2
B3
B4
9.00
6.75
4.50
2.25
0.00
log2(AUA+1)
Order in run

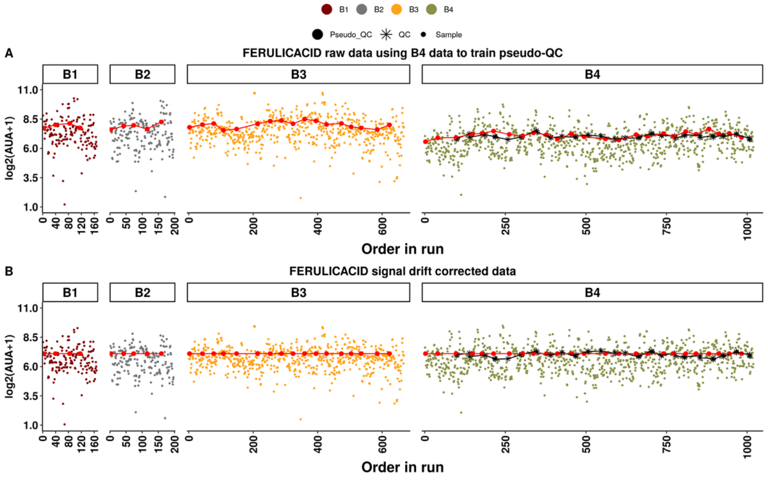

B1
B2
B3
B4
Pseudo_QC
QC
Sample
A
FERULICACID raw data using B4 data to train pseudo-QC
B
FERULICACID signal drift corrected data
log2(AUA+1)
11.0
8.5
6.0
3.5
1.0
0
40
80
120
160
50
100
150
200
400
600
250
500
750
1000
Order in run

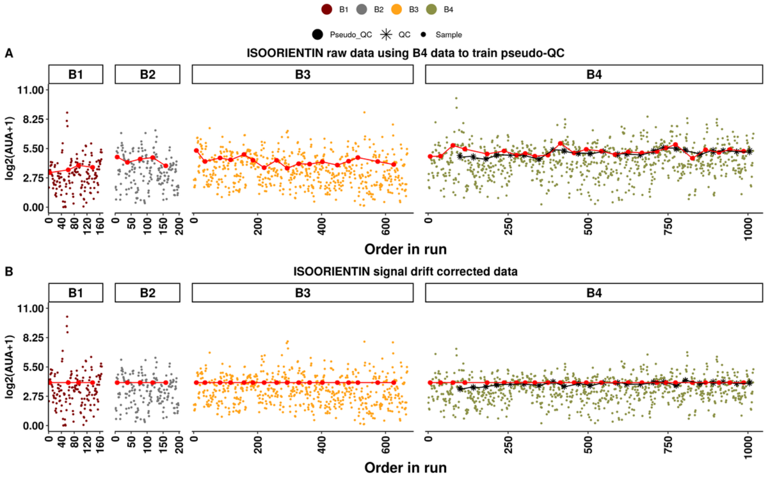

B1 B2 B3 B4
Pseudo_QC QC Sample
A
ISOORIENTIN raw data using B4 data to train pseudo-QC
B1
B2
B3
B4
log2(AUA+1)
11.00
8.25
5.50
2.75
0.00
0 40 80 120 160
0 50 100 150 200
0 200 400 600
0 250 500 750 1000
Order in run
B
ISOORIENTIN signal drift corrected data
B1
B2
B3
B4
log2(AUA+1)
11.00
8.25
5.50
2.75
0.00
0 40 80 120 160
0 50 100 150 200
0 200 400 600
0 250 500 750 1000
Order in run

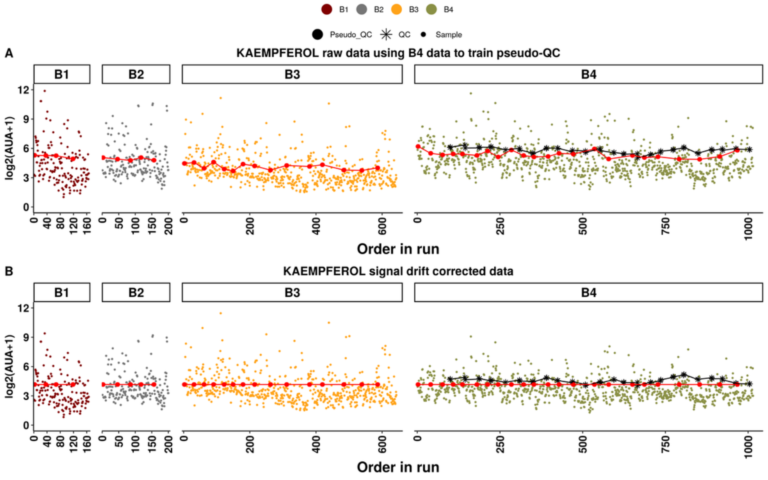
B1
B2
B3
B4
Pseudo_QC
QC
Sample
A
KAEMPFEROL raw data using B4 data to train pseudo-QC
log2(AUA+1)
Order in run
B
KAEMPFEROL signal drift corrected data
log2(AUA+1)
Order in run

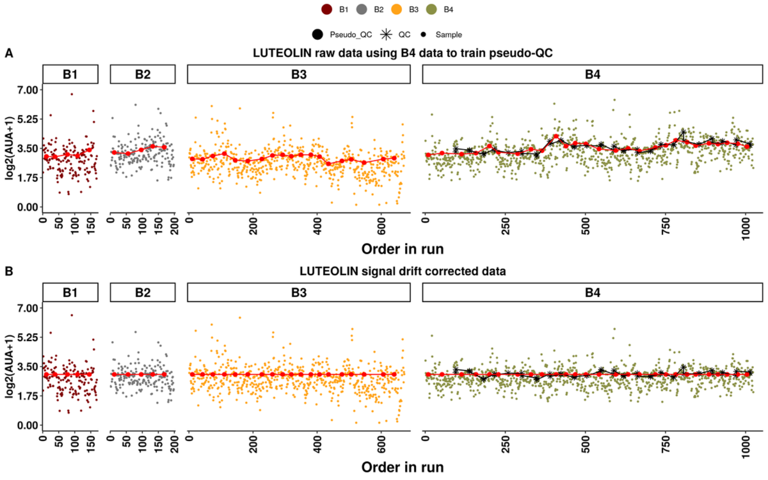

B1 B2 B3 B4
Pseudo_QC QC Sample
A
LUTEOLIN raw data using B4 data to train pseudo-QC
B1
B2
B3
B4
log2(AUA+1)
7.00
5.25
3.50
1.75
0.00
0 50 100 150
0 50 100 150 200
0 200 400 600
0 250 500 750 1000
Order in run
B
LUTEOLIN signal drift corrected data
B1
B2
B3
B4
log2(AUA+1)
7.00
5.25
3.50
1.75
0.00
0 50 100 150
0 50 100 150 200
0 200 400 600
0 250 500 750 1000
Order in run

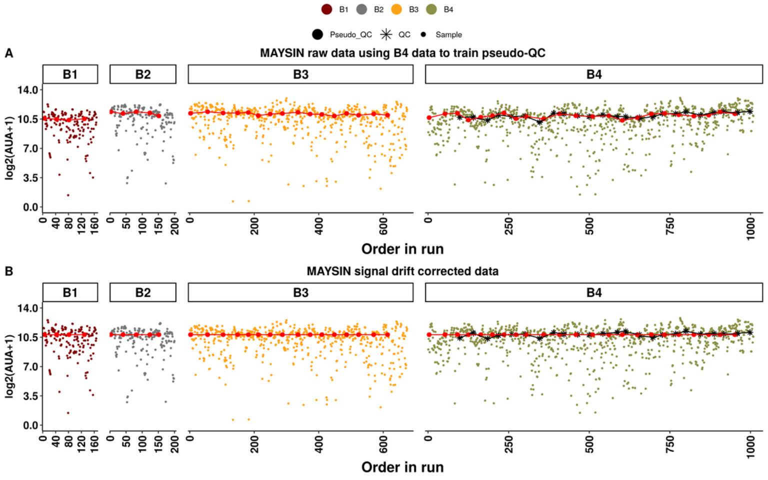

B1
B2
B3
B4
Pseudo_QC
QC
Sample
A
MAYSIN raw data using B4 data to train pseudo-QC
log2(AUA+1)
Order in run
B
MAYSIN signal drift corrected data
log2(AUA+1)
Order in run

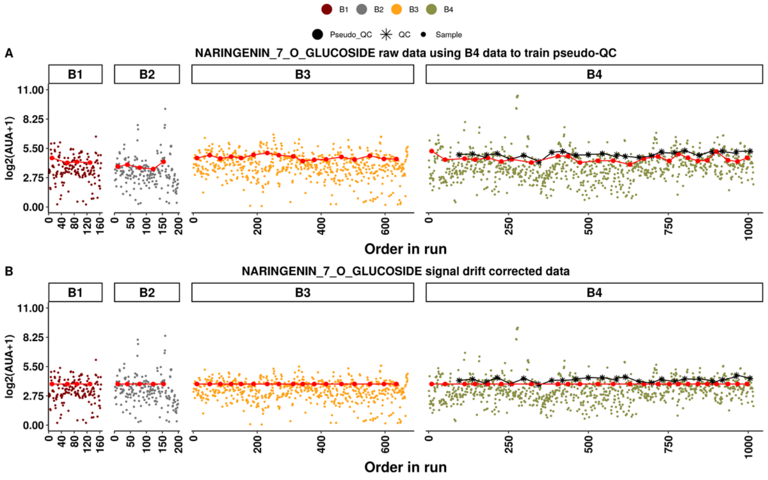

B1
B2
B3
B4
Pseudo_QC
QC
Sample
A
NARINGENIN_7_O_GLUCOSIDE raw data using B4 data to train pseudo-QC
log2(AUA+1)
11.00
8.25
5.50
2.75
0.00
0
40
80
120
160
50
100
150
200
400
600
250
500
750
1000
Order in run
B
NARINGENIN_7_O_GLUCOSIDE signal drift corrected data

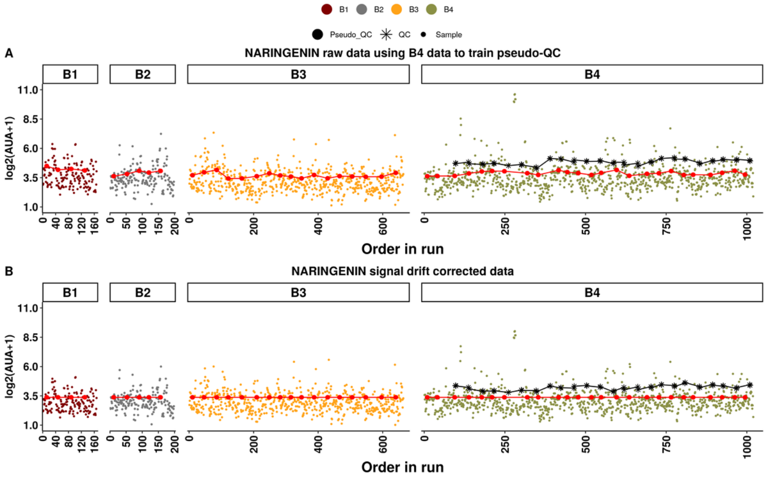
B1 B2 B3 B4
Pseudo_QC QC Sample
A
NARINGENIN raw data using B4 data to train pseudo-QC
B1
B2
B3
B4
log2(AUA+1)
11.0
8.5
6.0
3.5
1.0
0 40 80 120 160
0 50 100 150 200
0 200 400 600
0 250 500 750 1000
Order in run
B
NARINGENIN signal drift corrected data
B1
B2
B3
B4
log2(AUA+1)
11.0
8.5
6.0
3.5
1.0
0 40 80 120 160
0 50 100 150 200
0 200 400 600
0 250 500 750 1000
Order in run

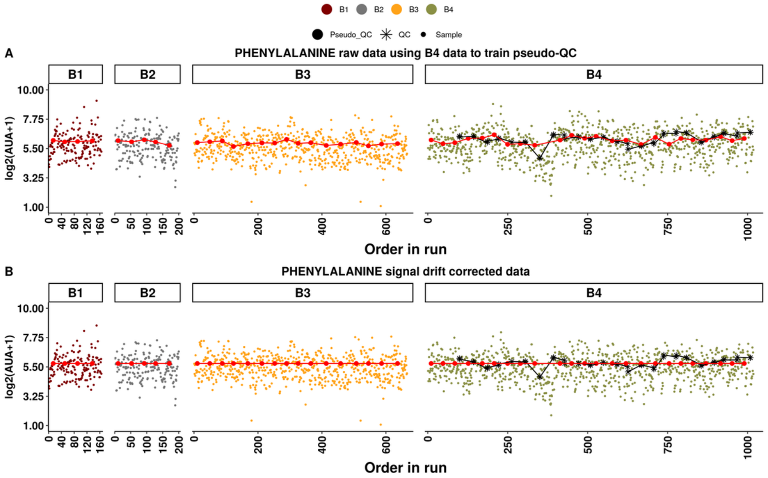

B1
B2
B3
B4
Pseudo_QC
QC
Sample
A
PHENYLALANINE raw data using B4 data to train pseudo-QC
B
PHENYLALANINE signal drift corrected data
log2(AUA+1)
10.00
7.75
5.50
3.25
1.00
0
40
80
120
160
0
50
100
150
200
0
200
400
600
0
250
500
750
1000
Order in run

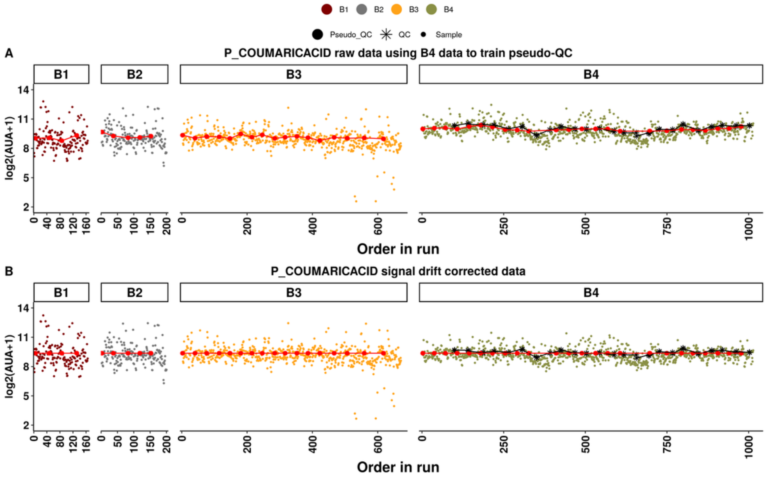

B1
B2
B3
B4
Pseudo_QC
QC
Sample
A
P_COUMARICACID raw data using B4 data to train pseudo-QC
log2(AUA+1)
Order in run
B
P_COUMARICACID signal drift corrected data
log2(AUA+1)
Order in run

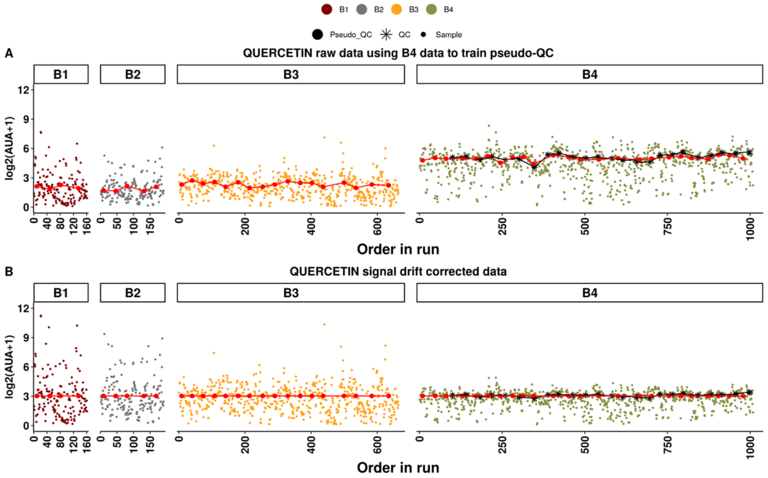

B1 B2 B3 B4
Pseudo_QC QC Sample
A
QUERCETIN raw data using B4 data to train pseudo-QC
B1
B2
B3
B4
log2(AUA+1)
Order in run
B
QUERCETIN signal drift corrected data
B1
B2
B3
B4
log2(AUA+1)
Order in run

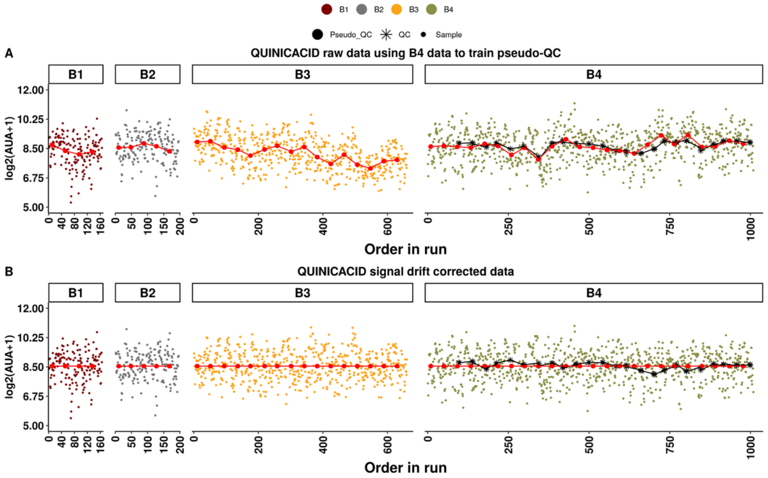

B1
B2
B3
B4
Pseudo_QC
QC
Sample
A
QUINICACID raw data using B4 data to train pseudo-QC
B
QUINICACID signal drift corrected data
log2(AUA+1)
12.00
10.25
8.50
6.75
5.00
Order in run
0
40
80
120
160
50
100
150
200
400
600
250
500
750
1000

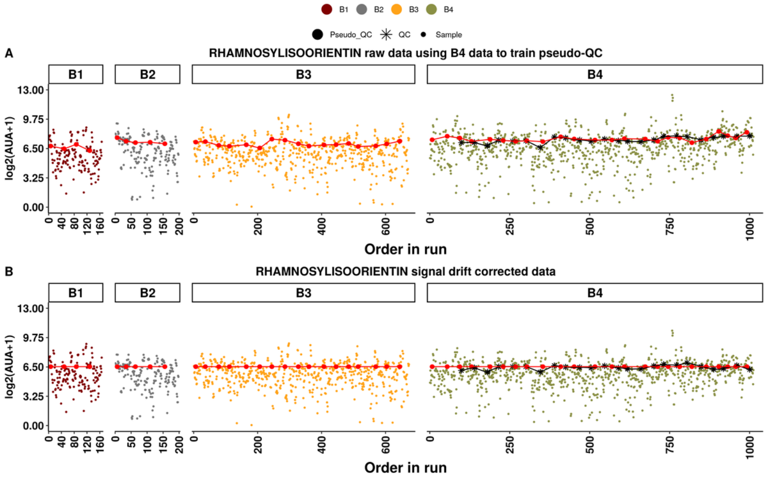

B1 B2 B3 B4
Pseudo_QC QC Sample
A
RHAMNOSYLISOORIENTIN raw data using B4 data to train pseudo-QC
B1
B2
B3
B4
13.00
9.75
6.50
3.25
0.00
log2(AUA+1)
0 40 80 120 160
0 50 100 150 200
0 200 400 600
0 250 500 750 1000
Order in run
B
RHAMNOSYLISOORIENTIN signal drift corrected data
B1
B2
B3
B4
13.00
9.75
6.50
3.25
0.00
log2(AUA+1)
0 40 80 120 160
0 50 100 150 200
0 200 400 600
0 250 500 750 1000
Order in run

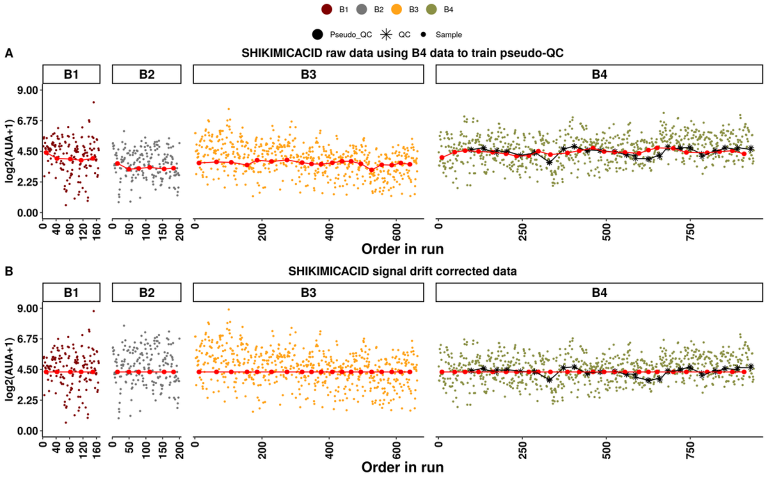
B1
B2
B3
B4
Pseudo_QC
QC
Sample
A
SHIKIMICACID raw data using B4 data to train pseudo-QC
B
SHIKIMICACID signal drift corrected data
log2(AUA+1)
Order in run

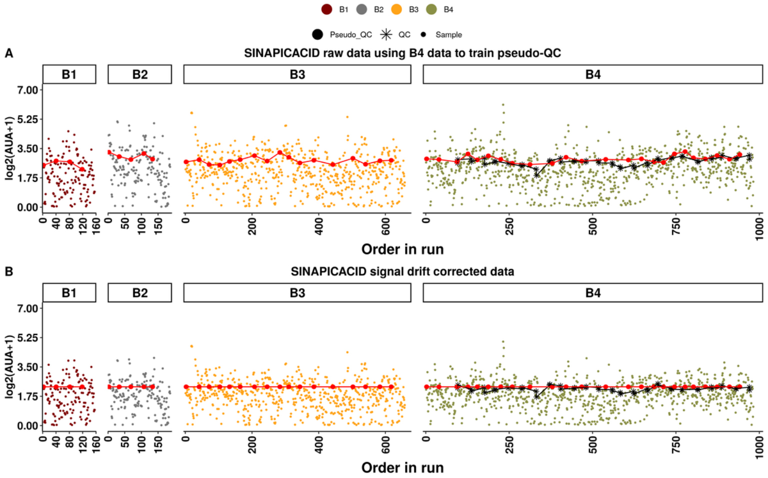
B1
B2
B3
B4
Pseudo_QC
QC
Sample
A
SINAPICACID raw data using B4 data to train pseudo-QC
B
SINAPICACID signal drift corrected data
log2(AUA+1)
7.00
5.25
3.50
1.75
0.00
0
40
80
120
160
0
50
100
150
0
200
400
600
0
250
500
750
1000
Order in run

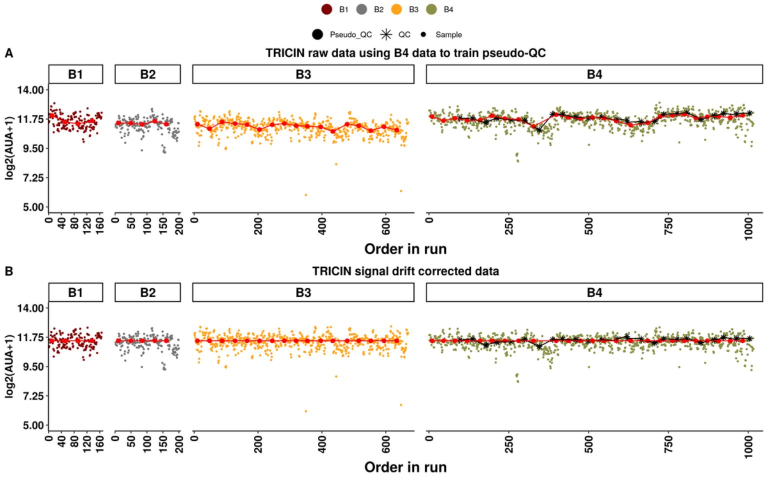

B1
B2
B3
B4
Pseudo_QC
QC
Sample
A
TRICIN raw data using B4 data to train pseudo-QC
log2(AUA+1)
14.00
11.75
9.50
7.25
5.00
0
40
80
120
160
50
100
150
200
200
400
600
250
500
750
1000
Order in run
B
TRICIN signal drift corrected data

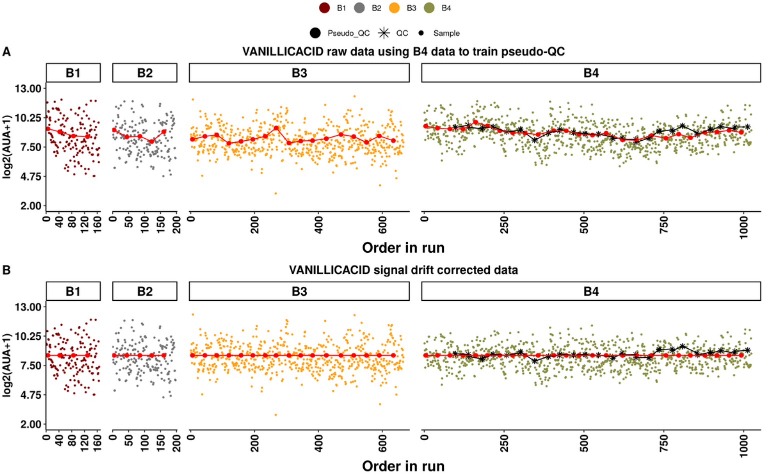

B1
B2
B3
B4
Pseudo_QC
QC
Sample
A
VANILLICACID raw data using B4 data to train pseudo-QC
B
VANILLICACID signal drift corrected data
log2(AUA+1)
Order in run
13.00
10.25
7.50
4.75
2.00
0
40
80
120
160
50
100
150
200
400
600
250
500
750
1000

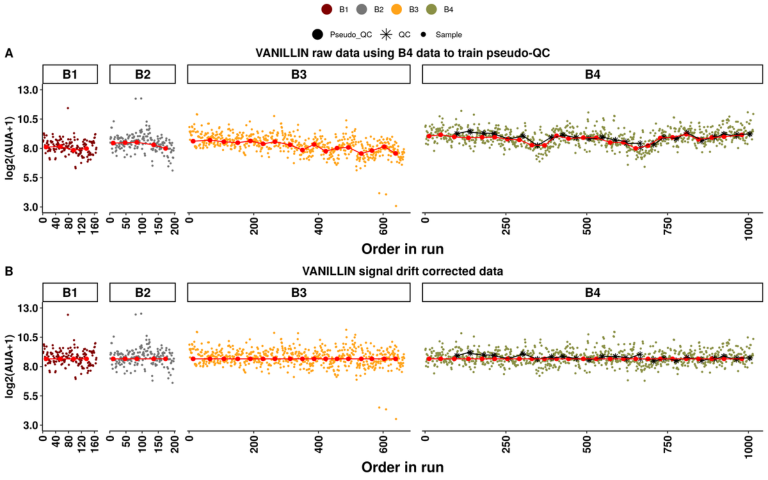
B1
B2
B3
B4
Pseudo_QC
QC
Sample
A
VANILLIN raw data using B4 data to train pseudo-QC
log2(AUA+1)
13.0
10.5
8.0
5.5
3.0
0
40
80
120
160
50
100
150
200
400
600
250
500
750
1000
Order in run
B
VANILLIN signal drift corrected data

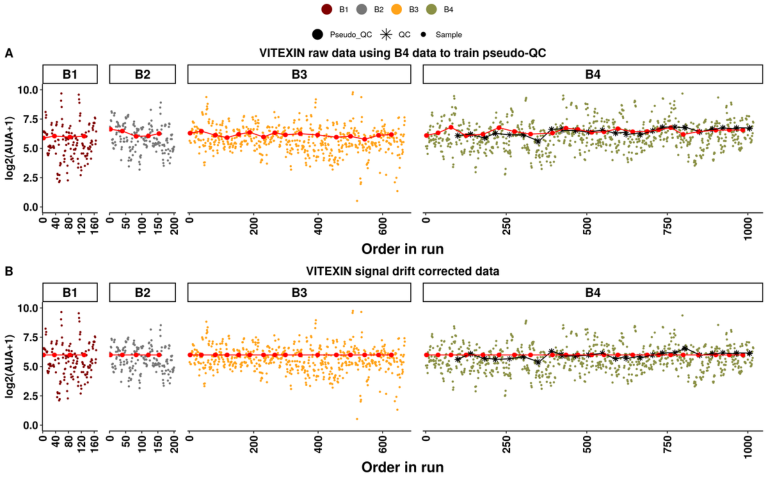

B1
B2
B3
B4
Pseudo_QC
QC
Sample
A
VITEXIN raw data using B4 data to train pseudo-QC
log2(AUA+1)
Order in run
B
VITEXIN signal drift corrected data
log2(AUA+1)
Order in run

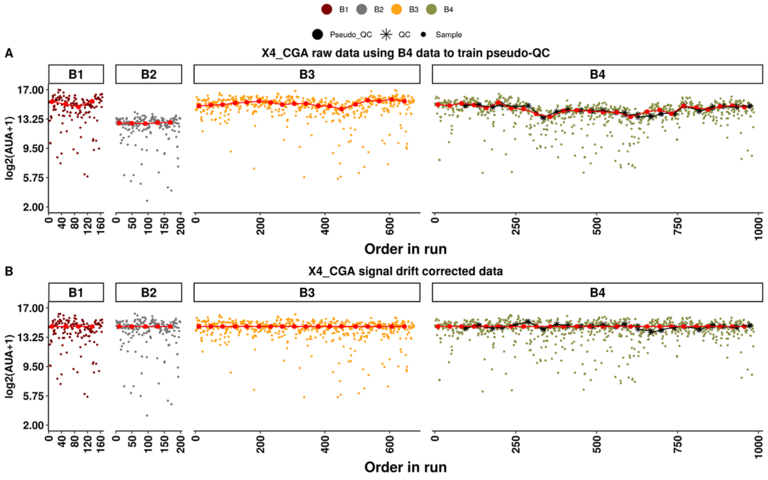
B1
B2
B3
B4
Pseudo_QC
QC
Sample
A
X4_CGA raw data using B4 data to train pseudo-QC
B1
B2
B3
B4
17.00
13.25
9.50
5.75
2.00
log2(AUA+1)
0 40 80 120 160
0 50 100 150 200
0 200 400 600
0 250 500 750 1000
Order in run
B
X4_CGA signal drift corrected data
B1
B2
B3
B4
17.00
13.25
9.50
5.75
2.00
log2(AUA+1)
0 40 80 120 160
0 50 100 150 200
0 200 400 600
0 250 500 750 1000
Order in run
